# CRISPR editing of CCR5 and HIV-1 facilitates viral elimination in antiretroviral drug-suppressed virus-infected humanized mice

**DOI:** 10.1073/pnas.2217887120

**Published:** 2023-05-01

**Authors:** Prasanta K. Dash, Chen Chen, Rafal Kaminski, Hang Su, Pietro Mancuso, Brady Sillman, Chen Zhang, Shuren Liao, Sruthi Sravanam, Hong Liu, Emiko Waight, Lili Guo, Saumi Mathews, Rahsan Sariyer, R. Lee Mosley, Larisa Y. Poluektova, Maurizio Caocci, Shohreh Amini, Santhi Gorantla, Tricia H. Burdo, Benson Edagwa, Howard E. Gendelman, Kamel Khalili

**Affiliations:** ^a^Department of Pharmacology and Experimental Neuroscience, Center for Neurodegenerative Diseases, University of Nebraska Medical Center, Omaha, NE 68198-5880; ^b^Department of Microbiology, Immunology, and Inflammation, Center for Neurovirology and Gene Editing, Lewis Katz School of Medicine at Temple University, Philadelphia, PA 19140; ^c^Department of Biology, College of Science and Technology, Temple University, Philadelphia, PA 19122

**Keywords:** HIV-1, long-acting ART, CRISPR-Cas9, CCR5 targeting, humanized mice

## Abstract

Treatment of HIV-1_ADA_-infected CD34+ NSG-humanized mice with long-acting ester prodrugs of cabotegravir, lamivudine, and abacavir in combination with native rilpivirine was followed by dual CRISPR-Cas9 C-C chemokine receptor type five (CCR5) and HIV-1 proviral DNA gene editing. This led to sequential viral suppression, restoration of absolute human CD4^+^ T cell numbers, then elimination of replication-competent virus in 58% of infected mice. Dual CRISPR therapies enabled the excision of integrated proviral DNA in infected human cells contained within live infected animals. Highly sensitive nucleic acid nested and droplet digital PCR, RNAscope, and viral outgrowth assays affirmed viral elimination. HIV-1 was not detected in the blood, spleen, lung, kidney, liver, gut, bone marrow, and brain of virus-free animals. Progeny virus from adoptively transferred and CRISPR-treated virus-free mice was neither detected nor recovered. Residual HIV-1 DNA fragments were easily seen in untreated and viral-rebounded animals. No evidence of off-target toxicities was recorded in any of the treated animals. Importantly, the dual CRISPR therapy demonstrated statistically significant improvements in HIV-1 cure percentages compared to single treatments. Taken together, these observations underscore a pivotal role of combinatorial CRISPR gene editing in achieving the elimination of HIV-1 infection.

## Significance

Long-acting antiretroviral drugs suppress viral replication in humanized mice. When administered CRISPR-mediated CCR5 and HIV-1LTR-Gag CRISPR editing the treatments resulted in the elimination of HIV-1 proviral DNA in lymphoid, bone marrow, and central nervous system tissues in 58% of the infected animals. Combinatorial CRISPR therapies proved statistically superior compared to single therapy for this HIV-1 cure strategy.

A functional cure of HIV-1 infection has been documented in three cases (1–3). In each, treatment of acute myeloid leukemia was performed with allogenic hematopoietic stem-cell transplants containing heterozygous or homozygous mutations in the HIV-1 chemokine C-C chemokine receptor type five (CCR5 Δ32) gene (4). HIV-1 cure included attenuation of spreading viral infection, presence of virus-infected cells and tissue reservoirs, and absence of latent integrated proviral DNA. Viral-rebound measurements were followed by the cessation of antiretroviral therapy (ART) (5–9). Current HIV-1 treatment includes both ART and broadly neutralizing antibodies (10). Both can reduce, but not eliminate infectious virus. HIV-1 establishes latency in CD4+ T cells and mononuclear phagocytes (monocytes, macrophages, and dendritic cells), which represents the primary barrier for cure. We posit that this barrier can be overcome by CRISPR-based gene editing. We previously demonstrated that elimination of viral infection could be achieved in HIV-1-infected CD34-NSG-humanized mice by long-acting ART and CRISPR-targeting HIV-1 LTR-Gag in a subset of infected animals (11). The current study was designed to improve upon the efficiency of viral elimination. This was investigated by inactivation of CCR5, a principal HIV-1 coreceptor followed by excision of HIV-1 LTR-Gag region from latent proviral DNA carrying infected cells. The use of two CRISPR formulations administered in a sequential manner was designed to block “infection spread” present at low levels during ART. This was followed by clearance of residual integrated HIV-1 DNA. The strategy was designed to affect viral elimination in HIV-1-infected mice (12–16).

## Results and Discussion

**Fig. 1.**
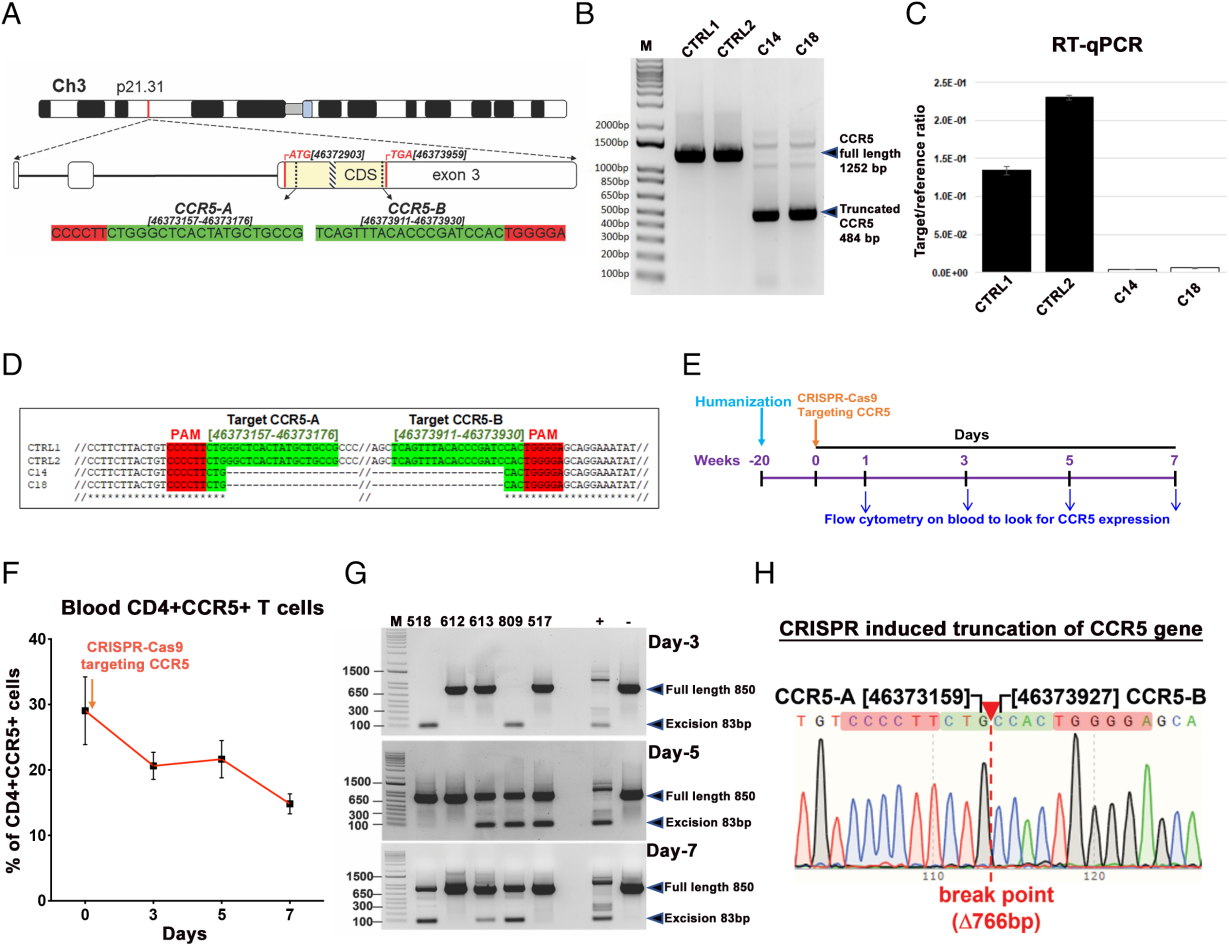
Guide-RNA location, CCR5 excision in vitro and in humanized mouse studies. (*A*) Chromosomal location and coordinates of CRISPR gRNA target sequences in the human *CCR5* gene. The *CCR5* coding sequence (CDS) is highlighted in yellow, positions of start and stop codons are in the red, the position of D32 mutation is shown as a patterned box, gRNA target sequences are highlighted in green, and PAMs in red. (*B*) Agarose gel analysis of PCR genotyping of CRISPR-Cas9-mediated cleavage of CCR5 gene. Genomic DNAs from two control (CTRL1 and CTRL2) and two CCR5 knockout (C14 and C18) TZM-bl single-cell clones were used as PCR template. (*C*) CCR5 mRNA expression in knockout clones was checked by reverse-transcription-qPCR from two controls and two knockout clones. (*D*) Alignment of Sanger sequencing results confirming CRISPR-induced truncation of CCR5. (*E*) Schematic illustration to look for CCR5 expression in human immune cells at different time points after a single IV injection of AAV_6_-CRISPR-Cas9-targeting CCR5 gene, in healthy humanized mice (n = 5). (*F*) Flow cytometric analysis of peripheral blood examined presence of CD3+CD4+CCR5+ T cells at 0, 3, 5, and 7 d after a single injection. Gating strategy was human CD45CD3CD4CCR5. Data are expressed as mean ± SEM. (*G*) CCR5 excision analysis on blood cells after single AAV_6_-CRISPR-Cas9 injection on the same humanized mice at different time points as described in *E* and * F*. The full-length and truncated bands are pointed in arrows. (*H*) Representative result of Sanger sequencing of the bands from humanized mice from *F* (mouse #809, day 3), confirming CRISPR-induced truncation of CCR5 gene.

We developed a pair of gRNAs that target specific regions within CCR5 (17) that includes the CCR5 delta32 deletion region using a bioinformatic approach that avoids any off-target effects (*SI Appendix*, Tables S1 and S2). We assessed its ability to edit two sites within the CCR5 gene and excise intervening DNA sequences (nucleotides 46373019 to 46373971 in Ch3, GRCH38.p14, Fig. 1*A*) using TZM-bl cells. Gene editing by CRISPR-Cas9 was confirmed by PCR genotyping, and the resultant truncated (484 bp) amplicons (Fig. 1*B*) were verified by Sanger sequencing (Fig. 1*D*). The absence of off-target cleavage at the top 10 in silico nominated off-target sites in the human genome for CCR5-A and CCR5-B gRNAs was verified by PCR-genotyping/Sanger sequencing analysis using genomic DNA derived from control (C1, C2) and CCR5-knockout (E14, E18) TZM-bl clones (*SI Appendix*, Tables S1 and S2 and Fig. S2). The lack of CCR5 expression (Fig. 1*C* and *SI Appendix*, Fig. S1*A*) protected CRISPR-treated cells from infection by CCR5-tropic HIV-1_BAL-GFP_ (*SI Appendix*, Fig. S1*B*). Next, the results were replicated in T lymphoid cell line HuTR5 (*SI Appendix*, Fig. S3). As expected, CRISPR-CCR5-AB-treated T cells showed resistance to infection with CCR5-tropic but not to infection with CXCR4-tropic or pantropic HIV-1 (*SI Appendix*, Fig. S3*D*). CCR5 is expressed on CD4+ T and myeloid lineage cells, serving as a principal means for HIV-1 cell entry. The expression may be altered without affecting immune function (17). To assess CRISPR gene editing for CCR5, we examined its expression in human immunocytes from control-uninfected humanized mice. Mice were injected with CRISPR-Cas9-targeting CCR5 delivered by AAV_6_ at 20 wk of age by a single intravenous (IV) tail vein dose (10^12^ GC units). Peripheral whole-blood cells were collected by the submandibular veinous puncture on days 1, 3, 5, 7, and examined by flow cytometry for CCR5 expression (Fig. 1*E* and *SI Appendix*, Fig. S4). Results demonstrated a decline in CCR5 expression levels in CD45+CD3+CD4+ T cells with a peak reduction at 7 d (Fig. 1*F*). The presence of CRISPR-induced truncated DNA was confirmed by PCR and genomic sequencing (Fig. 1 *G* and *H*). The data support the notion that CRISPR-Cas9 CCR5 could affect viral spread thereby reducing the target viral DNA for HIV-1 LTR-Gag-mediated excision (11).

**Fig. 2.**
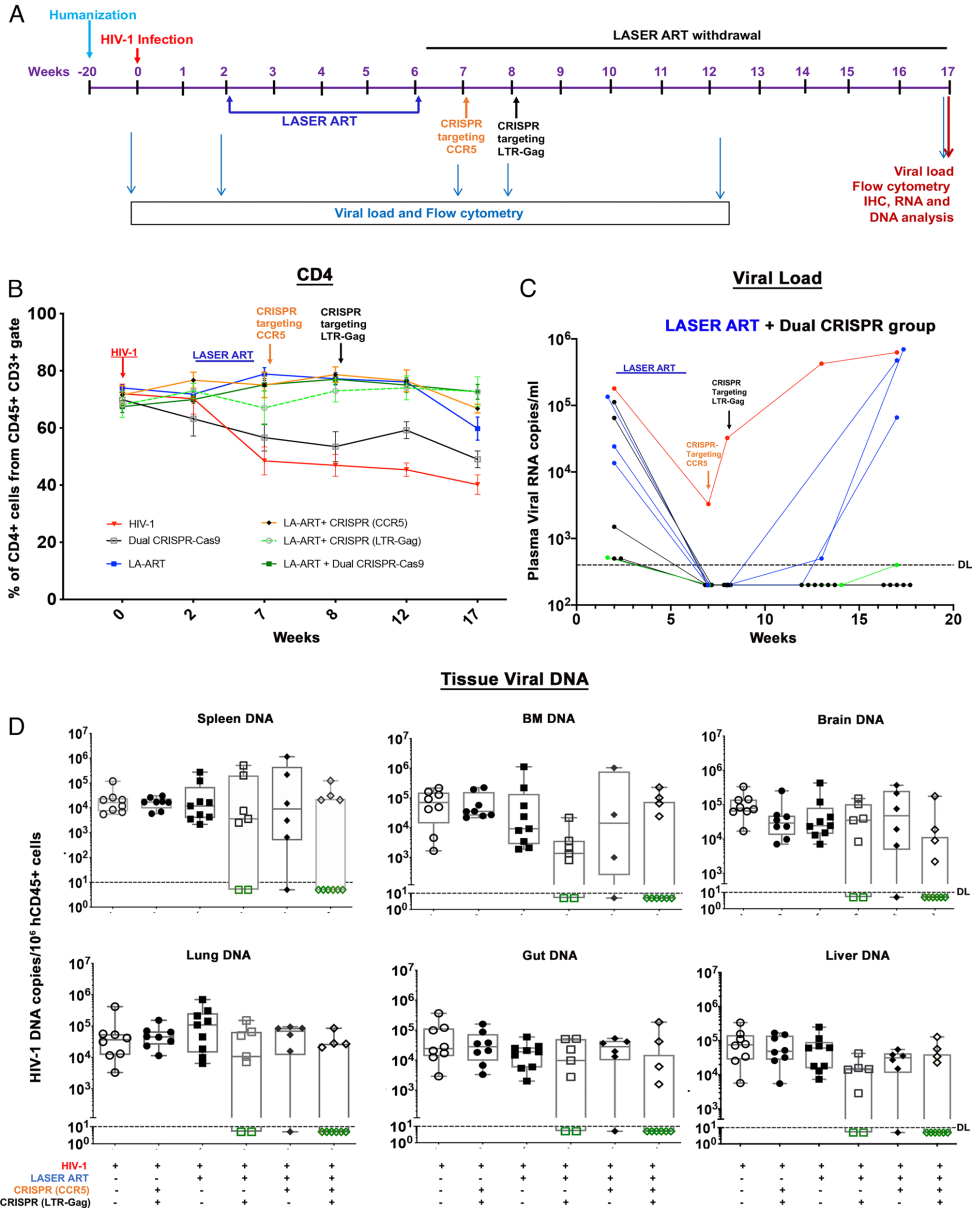
Viral and human immune profiles from LASER ART and CRISPR-Cas9 treatments of HIV-1-infected humanized mice. (*A*) Study scheme showing the timing of NSG-humanized mouse generation, HIV-1 infection, ART and single or dual CRISPR treatments to respective groups. After 2 wk of infection and confirmation of VL, mice were given intramuscular (IM) doses with 45 mg/kg NMCAB and NRPV and 40 mg/kg NM3TC, NMABC. Treatment was for 4 wk, followed by a single IV dose of AAV_6_-CRISPR-Cas9 CCR5, at week 7 and a second IV dose of AAV_9_-CRISPR-Cas9 LTR-Gag at week 8. Animals had antiretroviral medicines stopped for 11 wk at the time of sacrifice. (*B*) Evaluation of human CD45+CD3+CD4+ T cell numbers in humanized mice by flow cytometry tests on 0, 2, 7, 8, 12, and 17 wk postinfection. (Red line—HIV-1, Black—HIV+ dual CRISPR, blue—HIV+LASER ART, orange—HIV+LASER ART+CRISPR(CCR5), broken green—HIV+LASER ART+CRISPR(LTR-Gag) and solid green line indicates—HIV+LASER ART+ dual CRISPR group). (*C*) Plasma viral load assessment by determining viral RNA copies of individual animals assayed at 2, 7, 8, 12, and 17 wk after HIV-1_ADA_ infection from the LASER ART and dual CRISPR treatment group. One animal represented in green showed viral RNA at the detection limit at study end. (*D*) HIV-1 DNA was measured by semi-nested real-time qPCR assays. The data represent mean ± SEM for each group. Analyses were performed from spleen, BM, gut, brain, liver, and lung tissues in each of the treatment groups. Six out of ten animals with LASER ART+ dual CRISPR treatments, 2/7 in LASER ART + HIV CRISPR, and 1/6 in LASER ART + CCR5 CRISPR showed complete viral elimination from all analyzed tissues.

Following viral infection, we implemented a three-step treatment paradigm that included a LASER ART regimen of nanoformulated myristoylated cabotegravir (CAB) (18), lamivudine (3TC) (19), abacavir (20) (ABC), and native rilpivirine (RPV) (18–20) for suppression of viral replication followed by administration of AAV_6_/CRISPR-Cas9 for editing the CCR5 gene and mitigating viral spread, then removing targeted proviral DNA fragments by AAV_9_/CRISPR-Cas9 tailored to cleave the LTR-Gag region as illustrated in the experimental study scheme (Fig. 2*A*). To begin, humanized mice (hu-mice) were infected with 1.5 × 10^4^ tissue culture infective dose_50_ (TCID_50_) of HIV-1_ADA_. Plasma viral RNA were recorded at a median of 1.6 × 10^5^ ± 4.4 × 10^4^ copies/mL (Fig. 2*C*). Semi-nested real-time qPCR confirmed viral infection. Two-weeks following infection, animals were divided into six groups. The first received no treatment (n = 8); the second received both CCR5 and HIV-1 CRISPR-Cas9 at 1-wk intervals at 8 and 9 wk following viral infection (n = 8); the third received 40 to 45 mg/kg nanoformulated CAB, 3TC and ABC prodrugs, and RPV (n = 9); the fourth received an identical ART regimen followed by a single intravenous (IV) injection of AAV_9_-CRISPR-Cas9-targeting LTR-Gag (11, 21, 22) at week 8 (n = 7); the fifth group received the ART regimen and one single IV injection of AAV_6_-CRISPR-Cas9-targeting CCR5 (week 7, n = 6); and the sixth received ART and dual CRISPR (weeks 7 and 8, n = 10). Three uninfected mice were used as negative control. Animals were observed for 11 wk after ART cessation for evidence of viral rebound. Restoration of CD4^+^ T cell counts (72.6 ± 7) was observed in all ART-treated mice. CD4+ T cells were highest in group 6 as compared to group 3 animals (59.7 ± 11.5). Group 4 (70 ± 10) and 5 (66 ± 4) animals showed intermediate counts. In the absence of ART, group 2 animals, CD4+ T cell levels were low (44.3 ± 6) but modestly higher than those in group 1 (39%) (Fig. 2*B*). Viral rebound in plasma was not observed in five animals of group 6 (animal numbers 345, 354, 355, 365, and 373). One animal, number 392 had 400 HIV-1 RNA copies/mL that received ART and dual CRISPR-Cas9, two animals in the ART and CRISPR (LTR-Gag), (numbers 716 and 824), and one animal in the ART and CRISPR CCR5 group 5 (number 792) were found undetected (Fig. 2*C* and *SI Appendix*, Fig. S5). Statistical evaluations were performed employing log_10_ transformed datasets to reduce skewness. Comparisons of the different treatment groups showed significant treatment effects at 7, 8, 13, and 18 wk after viral challenge. One-way ANOVA tests of HIV-1-infected mice by Fisher LSD and of log_10_ transformed data performed at 18 wk showed a significant effect of the dual CRISPR treatments (*P *= 5.87 × 10^−5^). Controlling for false discovery rate (FDR) of 5% showed VL reductions in ART-treated HIV-1-infected mice given dual CRISPR (*P *= 0.0011) therapies compared against ART and single HIV-LTR-Gag (*P *= 0.004) and or single CCR5 (*P *= 0.0446) CRISPR treatments.

To affirm these datasets, viral DNA and RNA levels in infected mouse tissues were evaluated by semi-nested real-time qPCR (primers and probes) designed to detect HIV-1gag (11, 23). Combinatorial treatment (LASER ART and CCR5 and HIV-1 CRISPR) was more effective than either LASER ART or CRISPR-Cas9 alone in reducing viral DNA. The spleen, gut, bone marrow (BM), lung, liver, kidney, and brain from six of ten mice treated with ART and dual CRISPR therapies showed no viral DNA. This contrasted animal groups treated with ART and CRISPR HIV-1 (two of seven in group 4), and ART and CRISPR-CCR5 (one of six in group 5) (Fig. 2*D* and *SI Appendix*, Figs. S6 and S7). Similarly, combination of LASER ART and dual CRISPR-Cas9 treatments (group 6) demonstrated absence of viral RNA in the same six animal tissues (*SI Appendix*, Figs. S6 and S7).

**Fig. 3.**
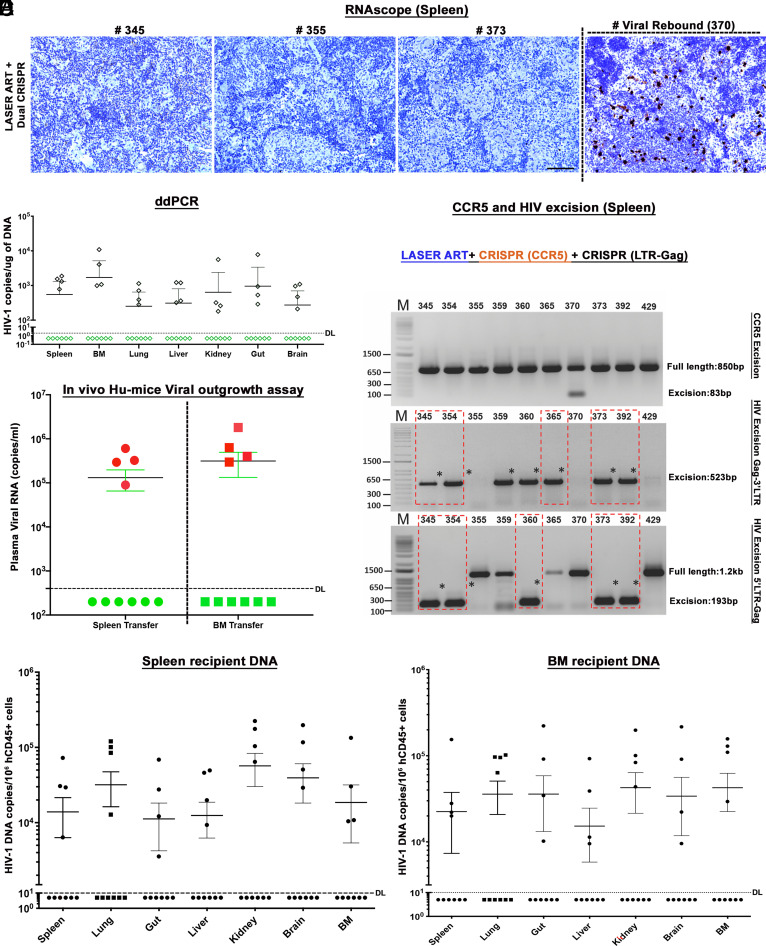
Viral elimination in HIV-1-infected and CRISPR-treated humanized mice. (*A*) RNAscope assay from representative LASER ART and dual CRISPR-treated mice showed no signals corresponding to the presence of viral RNA. In one mouse viral rebound was demonstrated at the study’s end. Human peptidylprolyl isomerase B was used as positive control for every tissue analyzed. Images are taken at 20× magnification. (*B*) Ultrasensitive ddPCR (detection limit of a single HIV-1 DNA copy) was used to assess viral DNA in organs of 10 mice treated with LASER ART and dual CRISPR. Note that in the same six animals with dual CRISPR treatment, complete elimination of virus was observed in all assayed tissues. (*C*) A VOA was performed by adoptive transfer of splenocytes and BM cells from LASER ART and dual CRISPR-Cas9-treated 10 animals to uninfected recipient CD34+ NSG-humanized mice. Cells isolated from same six animals failed to show viral recovery after 5 wk of examination by plasma viral RNA measurements as shown in green circles and boxes and used as the definition for viral eradication. (*D*) CCR5 and HIV-1 excision follows LASER ART treatment in infected humanized mice. Total DNA from spleen from 10 dual CRISPR-treated animals was tested for PCR genotyping with primer sets derived from the 5′LTR, 3′LTR, and the HIV-1 gag and CCR5 genes. The top panel shows excision of CCR5 DNA in HIV-1-infected humanized mice. Notably CCR5 expression returned to normal after 1 wk. In the latter case, an excised band was demonstrated in a single mouse. The middle panel shows excision of HIV-1 DNA following CRISPR-Cas9 3′LTR to Gag treatment in HIV-1-infected humanized mice and the bottom-panel shows CRISPR-Cas9 excision of HIV-1 Gag to 5′LTR in infected humanized mice. For adoptive transfer six animals failed efforts to recover HIV-1 DNA from recipient mice. Tests were performed from spleens by real-time semi-nested qPCR from spleen (*E*) and BM (*F*) by viral amplification. Six animals shown under dashed line in black dots had no virus recovered. These data confirmed complete viral elimination with no viral rebound from the donor cells. The detection limit of the detection assay was 10 copies. The data represent mean ± SEM for each group.

To affirm these results, HIV-1 RNA was examined by RNAscope. The assays were performed in 5-μm-thick spleen sections using an antisense probe designed to target 854-8291 HIV-1 base pairs. Under the current experimental conditions, the same six of ten mice in the dual CRISPR group and two of seven and one of six in the single CRISPR groups (HIV-1 and CCR5, respectively) showed no evidence of viral nucleic acid (Fig. 3*A* and *SI Appendix*, Fig. S8). Results from targeted qPCR for HIV-1 RNA corresponding to the pol gene (*SI Appendix*, Fig. S9) affirmed the test results in tissues from the same six mice. Further evidence for the absence of the HIV-1 DNA in the 6 dual CRISPR animals was provided through droplet digital PCR (ddPCR) tests, thus verifying prior results that no viral DNA was detected in the tissues from those same six animals (Fig. 3*B* and *SI Appendix*, Fig. S10). To further confirm, we adapted viral outgrowth assays (VOA) (11, 24) by adoptive transfer of BM cells and splenocytes from dual and single treatment groups to uninfected humanized mice (11, 24). No evidence of progeny virus recovery was observed in plasma (Fig. 3*D*) and tissues from recipients receiving splenocytes and BM cells from the same six infected animals that lacked evidence of viral DNA (Fig. 3 *E* and *F*) and RNA. Gel electrophoretic analysis of the PCR-amplified DNA fragments using specific pairs of primers designed for detection of the various cleavage events revealed robust cleavage and excision of viral DNA fragments obtained from the spleen (Fig. 3 *C*, *Bottom* two panels) of the group of animals treated with LASER ART and dual CRISPR-Cas9. Excision analysis to look for CCR5 truncation did not detect any bands (Fig. 3 *C*, *Upper*), as CCR5 and CXCR4 expression returned to baseline levels based on the expected repopulation of immune cells in the body and as confirmed by real-time qPCR from dual CRISPR-treated spleen tissues (*SI Appendix*, Fig. S11). Excision of the predicted viral DNA fragments in other tissues including the gut, liver, and brain was observed in most animals receiving dual treatments (*SI Appendix*, Figs. S12–S15) and in an animal that received single CRISPR-targeting LTR-Gag. The integrity and precision of the HIV-1 DNA excision by CRISPR-Cas9 were sequence verified. Off-target effects for CRISPR-HIV gRNAs were extensively analyzed in our previous study (11). The safety of gRNAs targeting the *CCR5* gene was verified using two CCR5k/o clones (C14 and C18, carrying complete biallelic Δ767bp CCR5-A to CCR5-B excision of the *CCR5* gene) and two control clones (Ctrl1 and Ctrl2) generated for this study in the TZM-bl cell line (Fig. 1 *B–D* and *SI Appendix*, Tables S1 and S2 and Fig. S2). No Indel mutations were detected at the investigated genomic locations across all the cellular clones tested, proving a lack of off-target activity of CCR5-A and CCR5-B gRNAs. The findings from the current study not only showed more than 50% HIV elimination from the ART and dual CRISPR-treated group but also reverified the HIV-1 elimination from a subset of ART and single CRISPR animals as shown previously (11).

**Fig. 4.**
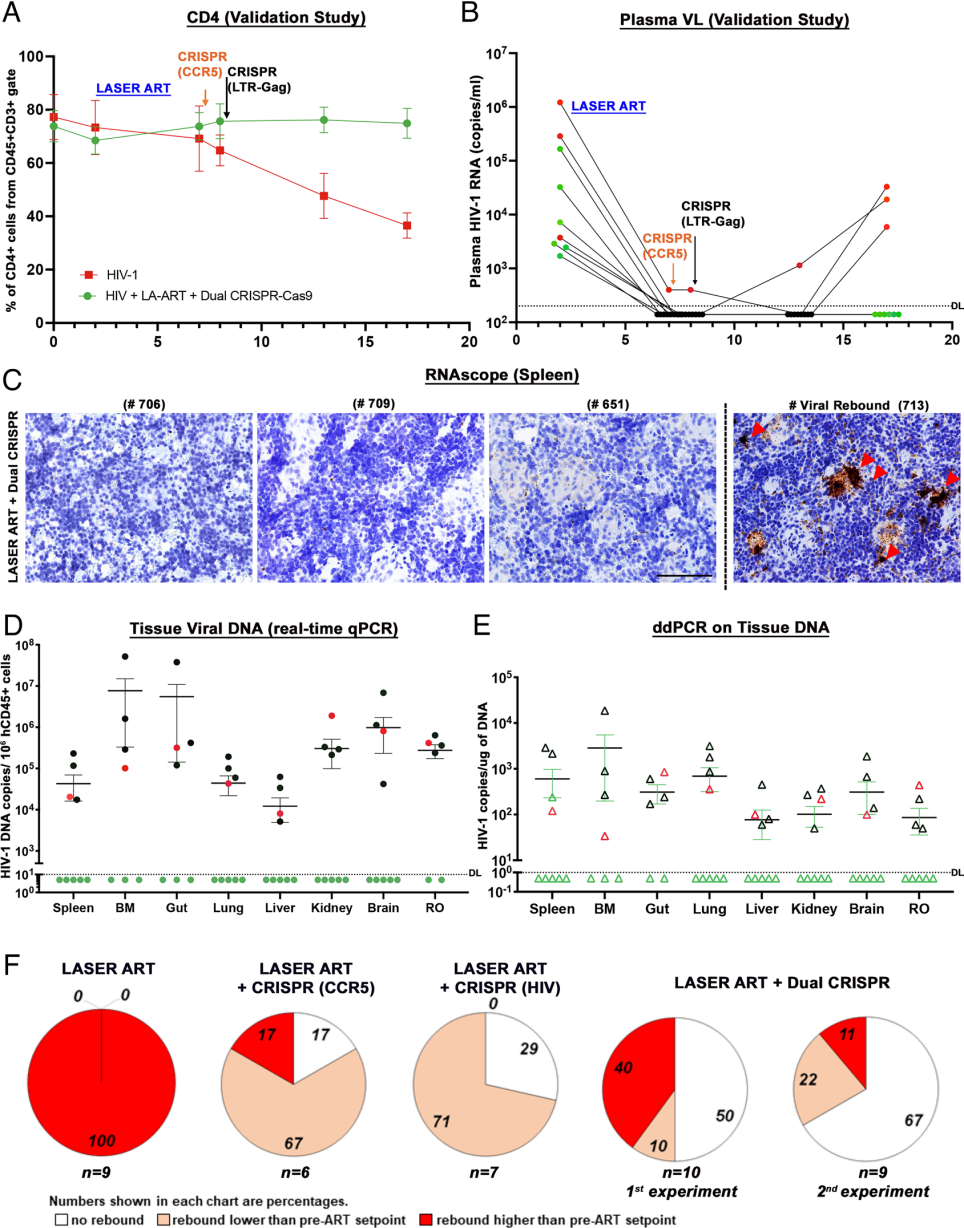
Viral and Immune profiles in HIV-1-infected and treated humanized mice of the validation study. (*A*) Flow cytometric evaluations of human CD4+ T cells in hu-mice assayed before (0) and 2, 7, 8, 13, and 17 wk postinfection from CD45+CD3+-gated populations. ART-treated and dual CCR5-HIV-1 CRISPR-administered mice showed restoration of absolute numbers of CD4+ T cells at the study end. (*B*) Plasma HIV-1 RNA copies of Hu-mice treated with ART and two sequential treatments of CRISPR-Cas9-targeting CCR5 and LTR-Gag indicated that six out of nine mice had no evidence of viral rebound in the plasma at 17 wk (study end). The sensitivity of detection was at 140 copies/mL after dilution factor adjustment. (*C*) Representative results from RNAscope assay revealed that 5 LASER ART and dual CRISPR-treated animals (numbers 706, 709, 622, 651, and 674) failed to demonstrate viral RNA amplification. The right panel shows one mouse spleen with viral rebound (#713). Images were captured at 40× magnification. (*D*) HIV-1 DNA analyses in the gag region using ultrasensitive semi-nested real-time qPCR assays from all the tissues of dual-treated animals (n = 9). Same five/nine animals shown in green closed circles showed no amplification of virus from all the tissues analyzed. The detection limit of the assay is 10 copies. One mouse (# 712) which was undetectable in plasma viremia was found to be HIV+ in tissue PCRs (shown in red closed circle). We could not find enough tissues from some mouse gut and BM for PCR, and the samples are missing from the datasets. The other three rebound mice (numbers 705, 707, and 713 shown in black circles) were found to be highly positive. The data represent mean ± SEM for each group. (*E*) ddPCR analysis of viral DNA from the nine dual-treated mice. The same five animals showed complete viral elimination in each of the tested tissues. One mouse (#712) which was undetectable in plasma viremia was found to be HIV-1 positive in tissue PCRs as shown in red open triangle. These results provide evidence of complete viral elimination in five mice (#s 706, 709, 622, 651, 674, green open triangle) and had no viral DNA detected in any tissues analyzed. (*F*) Plasma HIV-1 RNA copies of individual animals at 2WPI were compared to plasma HIV-1 RNA copies of the same animals at the study end, at 17WPI. The numbers of animals showing a lack of viral rebound (white), having rebound viral loads lower (salmon) or higher (red) than pre-ART setpoint are shown as a percentage for each of the ART-treated group. The numbers of animals in each category are indicated (*n* = *x*). To validate our findings in the ART and dual CRISPR treatment group (first evaluation) we conducted another independent experiment with a new set of nine humanized mice (second evaluation). Taken together, a total of 11 out of 19, ART and dual CRISPR-treated animals showed undetectable viral RNA at 17WPI (58%).

To further validate our observations, we performed replicate experiments with another independent set of humanized mice following a similar scheme as outlined in Fig. 2*A*. In this experiment, we infected 9 humanized mice with 2 × 10^4^ TCID_50_ of HIV-1_ADA_ for 2 wk. After infection was confirmed, LASER ART was administered for an additional 4 wk to minimize levels of spreading viral replication. Then the mice were treated with CRISPR-Cas9-targeting CCR5 (week 7) and LTR-gag (week 8) and evaluated evidence for viral rebound 9 wk after the last CRISPR administration (17 wk after viral infection). Six of nine (6/9) mice were found to have undetectable plasma viremia at study end (Fig. 4*B*). CD3+CD4+ T cells were restored in most of the animals (Fig. 4*A*). Viral DNA and RNA amplification from tissues by semi-nested real-time qPCR (Fig. 4*D*), RNAscope (Fig. 4*C*), and ddPCR (Fig. 4*E*) identified 5/9 animals (numbers 706, 709, 622, 651 and 674) without detectable HIV-1. Another animal (number 712) had undetectable plasma viremia; however, virus was found in tissues by ddPCR. This result highlighted the need to examine tissue HIV-1 latent reservoirs before concluding that virus was indeed eliminated. Humanized mouse VOA using spleen and BM tissues from dual-treated animals confirmed the absence of virus in plasma and tissues (*SI Appendix*, Figs. S16 and S17). PCR analysis of different tissues from the cured animal was performed and the excised bands were verified by sequencing. No ART or CRISPR-related toxicities were observed by histological analyses (hematoxylin and eosin staining) of spleen, liver, and kidney tissues (*SI Appendix*, Fig. S18). Throughout these studies, biodistribution of human cells in CRISPR-treated HIV-1-infected animals was tested by ddPCR (*SI Appendix*, Fig. S19). Distribution of adeno-associated virus (AAV) in CRISPR-treated HIV-1-infected mice was assessed by ultrasensitive ddPCR for analysis of Cas9 transgene DNA (*SI Appendix*, Fig. S20). ART and dual CRISPR treatment resulted in statistically significant increases in HIV-1 elimination (11 out of 19 hu-mice) compared against single treatments. By Fisher’s exact test, comparisons of ART, ART and CRISPR CCR5 (1/6 hu-mice, p=0.4) or ART and HIV-1LTR-Gag (2/7 mice, *P *= 0.175) showed no significant differences for viral elimination. However, combinatorial ART and CRISPR editing of CCR5, and HIV-1LTR-Gag resulted in significant increases in viral elimination in the two independent studies, thus demonstrating elimination of HIV-1 in 60% (6/10, *P *= 0.0108) and 56% (5/9, *P *= 0.0294) of the mice yielding an overall 58% HIV-1 elimination rate (11/19, *P *= 0.0039) in infected mice.

CRISPR gene editing by targeting host CCR5 and HIV-1 LTR-Gag while controlling viral replication by antiretroviral drugs can lead to HIV-1 elimination in tissue reservoirs of infected animals. Evidence was provided by the absence of viral rebound after 11 wk following ART cessation. These datasets were affirmed by sensitive PCR and viral rescue assays. We demonstrate that further improvements in LASER ART and CRISPR for combinatorial editing of viral and strategically important cellular genes such as CCR5 in hu-mice may achieve and serve as a proof-of-principle for further investigation toward clinical trials. Interestingly, analysis of plasma viral loads at the LASER ART start (2WPI) and withdrawal (17WPI) revealed an unanticipated effect of LASER ART and CRISPR treatments on viral rebound. In LASER ART only-treated animals at 17 WPI, plasma viral loads were higher (5× to 1,000×) than the initial setpoint (2 WPI). However, in the LASER ART + CRISPR-CCR5 and LASER ART + CRISPR-HIV-1 treatment groups most of the infected animals, 67% and 71%, respectively, showed virus levels lower than before treatment was initiated (Fig. 4*F*). These CRISPR-associated reductions in viral rebound may reflect reductions in the size of the reactivable viral reservoir due to resistance to new viral infections and/or direct damage to the intact proviral genomes. To achieve 100% HIV elimination, the CRISPR cargo carrying the excision agent needs to reach all the HIV-1 latently infected cells and compartments. AAV targeting is neither HIV-1 cell specific or targeted to any tissue compartments. Any latent viral compartments including the brain or lymphoid tissues where the AAV-CRISPR-cargo failed to reach then excise latent proviral DNA. This could have resulted in viral rebound in the remaining subpopulation (42%) of the dual-treated animals. In conclusion, despite the noted limitations, the sequential administration of combinations of two CRISPR-mediated antiretroviral strategies with ART achieved effective HIV-1 elimination and has potential for translation to the clinic.

## Materials and Methods

### Cell Culture and Reagents.

TZM-bl reporter cell line (NIH AIDS Reagent Program, Division of AIDS, National Institute of Allergy and Infectious Diseases, NIH, Bethesda, MD) and HEK-293T (ATCC, CRL-3216, Manassas, VA) cells were cultured in DMEM high glucose complemented with 10% fetal bovine sera (FBS) and gentamicin (10 µg/mL). HutR5 cells, human cell T-cell line stably expressing CCR5 (gift from Vineet N. Kewal Ramani, National Cancer Institute, Frederick) were cultured in Roswell Park Memorial Institute (RPMI) medium containing 10% FBS and gentamicin (10 ug/ml) (Sigma-Aldrich).

### Drugs.

CAB, 3TC, and ABC were purchased from tert-Butoxycarbonyl (BOC) Sciences. RPV was purchased from Hangzhou Bingo Chemical Co., Ltd. Poloxamer 407 (P407), N-2-hydroxyethylpiperazine-N'-2-ethanesulfonic acid (HEPES) buffer, ciprofloxacin, paraformaldehyde (PFA), and 3,3′-diaminobenzidine (DAB) were purchased from Sigma-Aldrich. Diethyl ether, endotoxin-free water, gentamicin, acetonitrile, methanol, KH_2_PO_4_, bovine serum albumin, Triton X-100, Liquid Chromatography Mass Spectrometry (LC-MS)-grade water, and TRIzol reagent were purchased from Fisher Scientific.

### Antibodies.

For flow cytometric analysis, we used a panel of antibodies (all from BD Biosciences) comprised of FITC-conjugated mouse anti-human CD45 (catalog #555482), Alexa Fluor 700-conjugated mouse anti-human CD3 (catalog #557943), Liquid Chromatography Mass Spectrometry (APC)-conjugated mouse anti-human CD4 (catalog #555349), and BV421-conjugated mouse anti-human CD8 (catalog #562428), PE-Cy7-conjugated mouse anti-human CD14 (catalog #555398), PE-Cy5-conjugated mouse anti-human CD19 (catalog #555414), and PE-conjugated mouse anti-human CCR5 (catalog #) antibodies. For immunohistochemical staining, we used monoclonal mouse anti-human HIV-1p24 (clone Kal-1, M0857, Dako, 1:10), monoclonal mouse anti-human leukocyte antigen (HLA-DR; clone CR3/43, Dako, 1:100), and the polymer-based HRP-conjugated anti-mouse EnVision and secondary antibodies were purchased from Dako. Peripheral blood was collected from the submandibular vein into ethylenediaminetetraacetic acid (EDTA)-coated tubes or by cardiac puncture at the study end. Blood leukocytes were tested for human pan-CD45, CD3, CD4, CD8, CD14, CCR5, and CD19 markers as seven-color combinations using LSR-II FACS analyzer (BD Biosciences). Antibodies and isotype controls were obtained from BD Pharmingen, and staining was analyzed with a FlowJo (BD Immunocytometry Systems). The gating strategy is as described previously (11). Results were expressed as percentages of total number of gated lymphocytes. The percentages of CD4- and CD8-positive cells were obtained from human CD3 + gate (11). We used absolute counts of human CD45 + cells to normalize each of the human cell datasets. Equivalent numbers of blood cells/mouse were used at each time point.

### Viral Stocks.

HEK-293T cells were transfected using the CaPO4 precipitation method in the presence of chloroquine (50 µM) and with 25 µg pNL4-3-BAL-GFP (gift from Christopher Aiken, Vanderbilt University Medical Center, Nashville) for 2.5 × 10^6^ cells/100 mm dish (to produce CCR5-tropic virus) or mixtures of 20 µg pNL4-3-GFP-P2A-Nef (13) and 5 µg pCMV-VSV-g (Addgene #8454) (to produce pan-tropic, VSV-g pseudotyped virus). Next day, media were replaced; and 24 and 48 h later, supernatants were collected, clarified at 3,000 rpm for 10 min, filtered through a 0.45-µm filter, and concentrated by ultracentrifugation for 2 h with a 20% sucrose cushion. Viral pellets were resuspended in Hank’s basic salt solution (HBSS) by gentle agitation overnight and aliquoted. Viral stocks were tittered by GFP-flow cytometry in TZM-bl cells. For infection experiments, weight (WT) and CCR5k/o TZM-bl cells were incubated with GFP reporter viruses at multiplicity of infection (MOI) 0.1-1 for 4 h in growth medium, then inoculum was removed, cells washed twice with phosphate-buffered saline (PBS), and fresh grow medium was added.

### Design and Cloning AAV-CRISPR-Cas9 Vectors.

The cloning of pX601-CMV-saCas9-LTR1-GagD AAV delivery vector was described previously (11). For targeting CCR5 the Benchling CRISPR guides designer tool (https://www.benchling.com) was used to screen sequence of the human *CCR5* (NCBI: NG_012637) gene for possible gRNA protospacer regions followed by saCas9-specific NNGRR(N) PAM. Next, a pair of gRNAs binding to coding sequence of the *CCR5* gene (CCR5-A and CCR5-B) was chosen based on the highest on-target cleavage score and the lowest nominated off-target cleavage score (strictly at least 4 to 5 mismatches compared with the target sequence thus making unwarranted cleavage at these sites highly unlikely). Similarly, as in the case of HIV-1, pairs of gRNAs were selected to induce 767-bp deletion in the coding sequence of the *CCR5* gene (Fig. 1*A*). Next, a pair of oligonucleotides with 5′-CACC and 3′-AAAC Bsa1 overhangs was obtained from Integrated DNA Technologies, annealed, phosphorylated, and ligated into *Bsa*I digested, dephosphorylated pX601-AAV-CMV: NLS-SauCas9-NLS-3xHA-bGHpA;U6::BsaI-sgRNA (a gift from Feng Zhang, Broad Institute of MIT and Harvard, Cambridge via Addgene) (61591; Addgene). For multiplex gRNA cloning, the target pX601-saCas9-CCR5B vector was linearized with *EcoR*I and KpnI, and the insertion fragment (U6-CCR5A-RNA scaffold) was produced via PCR using the primer pair T795/T796 with a mutation of the 3′-end KpnI site (for further addition of new sgRNAs) and the pX601-saCas9-CCR5A vector as the template. After purification, the linearized vector and the insertion PCR product were ligated using an In-Fusion HD Cloning Kit (Clontech). The positive duplex sgRNA-expressing pX601-saCas9-CCR5A+CCR5B clones were identified by double digestion with *Not*I and *BamH*I or *EcoR*I and verified by Sanger sequencing. Finally, sequence-verified plasmids were sent for packaging into the AAV-6 serotype (pX601-saCas9-CCR5A+CCR5B) or AAV-9-serotype (pX601-CMV-saCas9-LTR1-GagD) (UNC Vector Core, Chapel Hill, NC). The vendor provides viral titers as GC/mL. For CRISPR-CCR5 delivery, AAV6 serotype was chosen as it was shown to efficiently transduce primary cells of hematopoietic origin. AAV_9_ was chosen as the vector for CRISPR-HIV delivery for its robust transduction efficiencies in multiple tissues including the central nervous system as significant putative reservoirs for HIV-1. The notion was to permit efficient AAV entry into all putative HIV-1 target tissues including the brain.

### Generation of *CCR5*k/o Cell Lines.

TZM-bl cells were plated in 6-well plates at 1 × 10^5^ cells/well and cotransfected using Lipofectamine 2000 reagent (Invitrogen) with 1 µg control pX601-AAV-CMV::NLS-SaCas9-NLS-3xHA-bGHpA;U6::Bsa1-sgRNA (Addgene #61591) or 1 µg pX601-saCas9-CCR5A+CCR5B plasmid together with 0.2 µg pKLV-U6gRNA(Bbs1)-PGKpuro2ABFP (Addgene 50946) to provide puromycin selection marker. Next day, cells were transferred into 100-mm dishes and cultured in the presence of puromycin (Sigma) at concentration 1 µg/mL. After 2 wk, surviving clones were isolated using cloning cylinders (Corning) and after expansion, screened using CCR5-specific PCR-genotyping followed by verification of truncated CRISPR-cleaved/end-joined amplicons by Sanger sequencing (Genewiz). HutR5 cells were electroporated (Lonza Walkersville) with ribonucleoprotein complexes composed of recombinant SaCas9 (Aldevron) and synthetic CCR5-A and CCR5-B gRNAs (Synthego).

### Generation of Humanized Mice.

NOD.Cg-Prkdc^scid^ Il2rgt^m1Wjl^/SzJ (NSG) mice were obtained from the Jackson Laboratories, and bred under specific pathogen-free conditions in accordance with the ethical guidelines for care of laboratory animals at the University of Nebraska Medical Center (UNMC) set forth by the NIH. CD34+ cells were obtained from human cord blood and enriched using immune-magnetic beads (CD34+ selection kit; Miltenyi Biotec Inc.). CD34+ cell purity was >90% by flow cytometry. Cells were transplanted into newborn mice irradiated at 1 Gy using a RS-2000 X-ray Irradiator (Rad Source Technologies). Cell suspension was delivered by intrahepatic injection of 50,000 cells/mouse in 20 μL PBS with a 30-gauge needle. Humanization of the animals was affirmed by flow cytometry (25, 26) for CD45 and CD3 staining of blood immune cells.

### Study Approvals.

All experimental protocols involving the use of laboratory animals were approved by the UNMC Institutional Animal Care and Use Committee ensuring the ethical care and use of laboratory animals in experimental research. Human blood cells were isolated by leukapheresis from HIV-1/2 and hepatitis seronegative donors and were deemed exempt from approval by the Institutional Review Board (IRB) of UNMC. Human CD34 + hematopoietic stem cells were isolated from human umbilical cord blood and are exempt from UNMC IRB approval.

### Flow Cytometry.

GFP expression in infected cells was quantified using the Guava EasyCyte Mini flow cytometer (Guava Technologies). Cells were first fixed for 10 min in 2% PFA, then washed three times in PBS, and then analyzed. Peripheral blood was collected from the submandibular vein into EDTA-coated tubes or by cardiac puncture at the study end. Blood leukocytes were tested for human pan-CD45, CD3, CD4, CD8, CD14, CCR5, and CD19 markers as seven-color combinations using the LSR-II FACS analyzer (BD Biosciences). Antibodies and isotype controls were obtained from BD Pharmingen, and staining was analyzed with a FlowJo (BD Immunocytometry Systems). Results were expressed as percentages of total number of gated lymphocytes. The percentages of CD4- and CD8-positive cells were obtained from human CD3+ gate set (25–27).

### HIV-1 Infections.

Mice from a single donor were used for all dual treatment mice. Humanization of the animals was affirmed by flow cytometry (25, 26) for the presence of human CD45- and CD3-positive blood immune cells. At 18 to 20 wk of age, 48 NSG-hu mice were infected intraperitoneally with HIV-1_ADA_ (28) at 1.5 × 10^4^ TCID_50_/mL. Three control-uninfected animals were included in all test evaluations. Levels of viral RNA copies/mL were analyzed with the automated COBAS Ampliprep System V2.0/Taqman-48 system (Roche Molecular Diagnostics) (26, 27, 29). For this assay, 50 to 100 μL mouse plasma was diluted to 1 mL with sterile filtered normal human serum. The detection limit of the assay after dilution is 200 viral RNA copies/mL. Although the eclipse phase for viral infection in humans remains variable (30), the viral loads and CD4 + T cell depletion levels observed in our infected humanized mice are in point of fact reflective of the disease course in an infected human host. Indeed, only after weeks of infection we do observe significant cell loss (18, 23, 27, 31). These findings can be viewed as an affirmation of the model including CD4 + T cell-timed restorations seen after ART as is seen in humans. For the validation study, nine humanized mice were infected with HIV-1_ADA_ at 2.0 × 10^4^ TCID_50_/mL.

### Immunohistochemical Tests.

Spleen, lung, liver, and lymph nodes were perfused with PBS followed by 4% PFA and then postfixed overnight and embedded in paraffin. Five-micron-thick sections were cut from the paraffin blocks, mounted on glass slides, and labeled with mouse monoclonal antibodies (DakoCytomation) for HLA-DQ/DP/DR (clone CR3/43, 1:100) and HIV-1p24 (1:10). The polymer-based HRP-conjugated anti-mouse Dako EnVision system was used as a secondary detection reagent and developed with DAB. All paraffin-embedded sections were counterstained with Mayer’s hematoxylin. Deletion of primary antibodies or mouse IgG served as controls. Images were obtained with a Nikon DS-Fi1 camera fixed to a Nikon Eclipse E800 (Nikon Instruments) using NIS-Elements F 3.0 software.

### Preparation of Antiretroviral Nanoformulations.

Antiretroviral prodrugs and their polymer encasements were performed as previously described (18–20). Myristoylated modifications for CAB, 3TC, and ABC were made (NMCAB, NM3TC, and NMABC) then transformed into surfactant stabilized nanoparticles. RPV was encased solely by P407 in unmodified form (29) to form crystalline nanoformulated drugs. Particle size, polydispersity index, and zeta potential were determined by dynamic light scattering using a Malvern Nano-ZS (Malvern). Final drug concentrations in the nanoformulation suspensions and injection solutions were determined by high-performance liquid chromatography ultraviolet/ visual (HPLC-UV/Vis) and ultra-high performance liquid chromatography tandem mass spectrometry (UPLC-MS/MS). A 40 to 50-µL volume for each nanoformulation (NMCAB/NRPV and NM3TC/NMABC) was administered by intramuscular injection in opposing thigh muscles of the mice.

### Nucleic Acid Extractions and q-PCR Assays.

In studies presented in Figs. 2–4 and Supporting figures, total viral nucleic acids (RNA and DNA) were extracted from the spleen, BM cells, lung, gut, liver, kidney, and brain using a Qiagen Kit (Qiagen) according to the manufacturer’s instructions. Total cellular DNA obtained from the HIV-1-infected cell line ACH2 served as a positive control and standards, while human genomic DNA obtained from uninfected NSG-hu mice served as a negative control. Cell-associated HIV-1 RNA and DNA were quantified by real-time qPCR and ddPCR assays. Because of extremely low numbers of latently infected human cells in HIV-infected NSG-hu mice after long-term ART, detection of total HIV-1 DNA, required two rounds of PCR amplification. The first round of PCR was performed on a conventional PCR machine (T100 Thermal Cycler, Biorad) in 25 μL of PCR reaction mix containing 500 ng of template and 50 ng each of both primers annealing to the HIV-1 gag region, and the reaction conditions are as follows: 94 °C for 3 min, followed by 15 cycles of 94 °C for 30 s, 55 °C for 30 s, and 72 °C for 1 min. The product of the first PCR was subsequently used as a template in the second semi-nested real-time PCR amplification performed on the ABI Step One Plus real-time PCR machine (Applied Biosystems) using TaqMan detection probe and primers (23, 25). Two microliter of the first PCR product was diluted to 50 μL with PCR master mix containing two primers at 0.2 μM each and a 0.2 µM TaqMan dual-labeled fluorescent probe. Real-time PCR settings were as follows: 50 °C for 2 min, then 95 °C for 10 min, followed by 40 cycles of 95 °C for 15 s, and 60 °C for 1 min. The amplicon sizes are 221 bp for the first round of PCR and 83 bp for the second round (real-time) PCR. DNA extracted from ACH2 cells containing one integrated copy of HIV-1 per cell was used as standard in serial 10-fold dilutions with HIV copy numbers ranging from 10^1^ to 10^5^ DNA copies/reaction (23). Human CD45 species-specific primers and probes were obtained from Thermo-Fisher Scientific (cat. no. 433182 for Hs0036534_g1). For viral excision testing, frozen tissues sent to Temple University from the UNMC were homogenized using Bullet Blender homogenizer (Next Advance) using bead combinations and settings specific for every tissue according to the manufacturer’s protocols. T1 buffer from NucleoSpin Tissue kit (Macherey-Nagel) was used for homogenization/initial lysis followed by overnight proteinase K digestion. Extraction of genomic DNA was completed according to the manufacturer’s protocol. For standard PCRs as described (29), 500 ng of extracted DNA was subjected to PCR using the Fail Safe PCR kit and buffer D (Epicentre) under the following PCR conditions: 94 °C 5 min, 30 cycles (94 °C 30 s, 55 °C 30 s, 72 °C 30 s), 72 °C 7 min using first round primers followed by nested PCR using diluted first round PCR reaction. Nested PCR products were subjected to Sanger sequencing directly if only one amplicon population was detected by agarose gel electrophoresis. For multiple amplicons detected, and to investigate the composition of HIV-1 excision, each amplicon population was separated and purified from an agarose gel electrophoresis and then cloned into TA vector (Invitrogen). Plasmid DNA containing excised HIV amplicon was purified from each bacterial colony for Sanger sequencing (Genewiz). Sanger sequencing results were analyzed using the Clustal Omega (European Molecular Biology Laboratory (EMBL)-EBI) multiple sequence alignment tool.

### ddPCR Detection.

ddPCR was performed based on the water–oil emulsion droplet technology with probes prepared in the QX200™ Droplet Digital™ PCR system (Bio-Rad Laboratories). The genomic DNA isolated from different tissues was PCR-amplified using Taqman sets specific to human and mouse Tert genes, and the human/mouse Tert signal ratios were plotted as % of human cells in mouse. For AAV vector biodistribution Taqman sets specific to saCas9 gene sequence was present in all AAV-CRISPR vectors used in the study. Human Tert served as a reference for human cell copy number in samples. A total of 100 to 200 ng DNA from each tissue was used as template for ddPCR amplifications with the same thermal cycling conditions used for real-time q-PCR detection. Data acquisition and analysis were done using QX200 droplet reader and QuantaSoft™ software provided with the instrument.

### RNAscope.

Viral RNA was detected as single brown dots or cluster of dots in 5-μm-thick paraffin embedded spleen and lymph node tissue sections using antisense probe V-HIV1- Clade-B (Cat no 416111) targeting 854–8291 bp of HIV-1_NL4–3_ (32). Human peptidylprolyl isomerase B (PPIB) was used as positive control for the spleen tissue analyzed (images were captured at 20-× magnification). All reagents are from Advanced Cellular Diagnostics, Newark, CA.

### Adoptive Transfer.

Splenocytes and BM cells (8–10 × 10^6^) were harvested at the time of sacrifice from NSG-hu mice that were HIV-1_ADA_ infected with LA-ART and single or dual CRISPR-Cas9. The cells were adoptively transferred into unmanipulated 18 to 20-wk-old CD34 + HSC-NSG mice as described (11, 24). Cell counts and viability tests were determined by both trypan blue and live/dead stains on the TC-20 automated cell counter (Bio-Rad). Cells were injected IP into mice and monitored for an additional 5 wk. These experiments were performed to cross validate eradication of viral infection that could occur from latent reservoirs and not detected by either qPCR, RNAscope, and ddPCR assays. Viral load was measured from blood samples of the adoptively transferred mice using automated COBAS Ampliprep System V2.0/Taqman-48 system, and immune cell profiles (CD4 and CD8 + T cells by flow cytometry) recorded, in parallel. Residual virus from all humanized mouse tissues was examined by qPCR and ddPCR assays. Virus was not detected in plasma or tissues from six LA-ART and dual CRISPR-treated and adoptively transferred animals as shown in Fig. 3 *D*–*F*.

### Sequence and Statistical Analyses.

Sanger sequencing results were analyzed using the Clustal Omega (EMBL-EBI) multiple sequence alignment tool (for fasta files) and SnapGene Viewer (Dotmatics) (for. ab sequencing chromatogram files). Data were analyzed by GraphPad Prism 8.0 software (La Jolla, CA, USA). Data are represented as the mean ± SEM. Experiments were performed using a minimum of three biologically distinct replicates. For comparisons of two groups, Student’s *t* test was used. T cell populations, viral RNA and DNA, and viral load were analyzed by one-way ANOVA with Bonferroni correction for multiple comparisons. For studies with multiple time points, two-way factorial ANOVA and Bonferroni’s post hoc tests for multiple comparisons were performed. Multiple comparisons were corrected for the FDR using the Benjamini–Hochberg procedure. Animal studies included a minimum of five to seven animals per group unless otherwise noted. Extreme outliers beyond the 99% CI of the mean and threefold greater than the SEM were excluded. Significant differences were determined at a *P* < 0.05.

## Supplementary Material

Appendix 01 (PDF)

## Data Availability

All study data are included in the article and/or *SI Appendix*.
