# Supplementary material for: CRISPR editing of CCR5 and HIV-1 facilitates viral elimination in antiretroviral drug-suppressed virus-infected humanized mice
Source: Proc Natl Acad Sci U S A. 2023 May 1;120(19):e2217887120. doi: 10.1073/pnas.2217887120 (PMC10175831; doi:10.1073/pnas.2217887120)
Supplement: Supplementary file 1 — Appendix 01 (PDF) [file pnas.2217887120.sapp.pdf]

Supporting Information

CRISPR Editing of CCR5 and HIV-1 Facilitates Viral Elimination in Antiretroviral Drug-Suppressed Virus-Infected Humanized Mice

Prasanta. K. Dash[1†], Chen Chen[2†], Rafal Kaminski[2†], Hang Su[1†], Pietro Mancuso[2], Brady Sillman[1], Chen Zhang[1], Shuren Liao[2], Sruthi Sravanam[1], Hong Liu[2], Emiko Waight[1], Lili Guo[1], Saumi Mathews[1], Rahsan Sariyer[2], R Lee Mosley[1], Larisa Poluektova[1], Maurizio Caocci[2], Shohreh Amini[2,3], Santhi Gorantla[1], Tricia H. Burdo[2], Benson Edagwa[1], Howard E. Gendelman[1*], Kamel Khalili[2*]

[1]Department of Pharmacology and Experimental Neuroscience, University of Nebraska Medical Center, Omaha, NE 68198-5880 USA

[2]Department of Microbiology, Immunology, and Inflammation/Center for Neurovirology and Gene Editing, Lewis Katz School of Medicine at Temple University, Philadelphia, PA 19140 USA

[3]Department of Biology, College of Science and Technology, Temple University, Philadelphia, PA 19122 USA

[†]Equal contribution by these authors

Short Title: HIV-elimination using Dual CRISPR and long-acting ART

***Corresponding authors: Howard E. Gendelman, MD**, Department of Pharmacology and Experimental Neuroscience, University of Nebraska Medical Center, Omaha, NE; email: ; phone: 402 559 8920; **Kamel Khalili, PhD**, Department of Microbiology, Immunology and Inflammation/Center for Neurovirology and Gene Editing, Lewis Katz School of Medicine at Temple University, Philadelphia, PA; kamel.khalili@temple.edu; phone: 215 707 5192

Supporting Data Table Figures with Legends

Table 1. OFF-target analysis for CCR5-A gRNA.

Sequence	PAM	Score	Gene	Chromosomal location	Strand	Mismatches	On-target	PCR/Sanger sequencing	InDels presence
GCGGCAGCATAGTGAGCCCAG	AAGGG	**100**	**CCR5**	chr3:-46373160	-1	0	TRUE		
GGGGCTGCCTGGTGAGCCCAG	AGGGG	1.5		chr2:-10061501	-1	4	FALSE	YES	ND
AGAGCAGCCCAGTGAGCCCAG	TGGGG	1		chr3:-14166014	-1	5	FALSE	YES	ND
CTGGAAGCTAAGTGAGCCCAG	GTGAG	1		chr5:+74140777	1	5	FALSE	YES	ND
CGGACAGCAGCGTGAGCCCAG	CGGGA	0.9		chr12:+125091629	1	5	FALSE	YES	ND
GTGGCTGCAGTGTGAGCCCAG	CAGAG	0.9		chr7:+157772472	1	4	FALSE	YES	ND
GCCACAGCTTAGGGAGCCCAG	CTGAG	0.8		chr3:-134084956	-1	4	FALSE	YES	ND
CTGGCAGTGCAGTGAGCCCAG	CGGGG	0.6	DMBX1	chr1:-46512282	-1	5	FALSE	YES	ND
CCGACTGAAGAGTGAGCCCAG	CAGAA	0.6	BCL2L1	chr20:+31665973	1	5	FALSE	YES	ND
AGGGCAGTATCCTGAGCCCAG	TGGAG	0.6		chr5:+133754764	1	5	FALSE	YES	ND
TCTGCAGTATTATGAGCCCAG	CAGAA	0.6		chr6:+56448824	1	5	FALSE	YES	ND
CCTGGAGAATAGAGAGCCCAG	GAGGG	0.5		chr6:+47146660	1	5	FALSE		
ACAGCGGAATAGGGAGCCCAG	GAGAG	0.5	OPN4	chr10:-86660021	-1	5	FALSE		
GGGGTAGCATATTGAGCCCAC	CTGGG	0.5		chr6:-86940192	-1	4	FALSE		
CCAGGAACATAGTGAGCCCAC	AGGAA	0.5		chr19:+45701655	1	5	FALSE		
TGGGCGGCAGAGGGAGCCCAG	GGGAG	0.5		chr7:+155382566	1	5	FALSE		
GGGGCAGCACTGTGAGCCCAA	GGGGA	0.5		chr20:+32697572	1	4	FALSE		
GCGGCGCCAAGGTGAGCCCAG	CTGGA	0.5	ALDH2	chr12:-111783206	-1	4	FALSE		
GCAGCAGCTGAGGGAGCCCAG	AAGGA	0.5	IMP5	chr17:+45845624	1	4	FALSE		
AGGGCAGCAAGGGGAGCCCAG	GCGGG	0.5	TSKU	chr11:-76797375	-1	5	FALSE		
GATGCAGCAGAGTGAGCCCTG	AGGAA	0.4		chr2:-149031578	-1	4	FALSE		
GGGGCTGCAGAGTGAGCCCTG	ATGGA	0.4		chr10:-77895107	-1	4	FALSE		
CAGGCAGCCCAGTGAGCCCCG	CTGGG	0.4		chr15:+45466875	1	5	FALSE		
GCTGCAGCTGAGTTAGCCCAG	GTGAG	0.4		chr1:-55049959	-1	4	FALSE		
GCTGGACCATAGTCAGCCCAG	CTGGA	0.4		chr3:-133897752	-1	4	FALSE		
ACGGGAGGATTGTCAGCCCAG	ATGGG	0.4		chr1:+33247763	1	5	FALSE		
CCGGCAGCTCTGGGAGCCCAG	CGGAG	0.4		chr14:-36577066	-1	5	FALSE		
ACAGCAGCTCAGTGAGCCCTG	CGGGA	0.4		chr16:-88478547	-1	5	FALSE		
AGGGCAGGAGAATGAGCCCAG	TGGAG	0.4		chr2:-585678	-1	5	FALSE		
GCAGCATAAAAGTGAGCCCAG	ATGAG	0.4		chr4:+120639359	1	4	FALSE		
CCTGCAAGAGAGTGAGCCCAG	GAGGA	0.4		chr9:+36942849	1	5	FALSE		
CCAGCTGCATAGAGAGCTCAG	CTGGG	0.4		chr5:+141788833	1	5	FALSE		
CCAGCAGGAAAGAGAGCCCAG	TTGGG	0.3		chr18:+26780609	1	5	FALSE		
AGGGCAGGATACAGAGCCCAG	AAGGA	0.3		chr13:+99614149	1	5	FALSE		
TAGGAAGCATTGTGAGGCCAG	GTGAG	0.3		chr19:-437930	-1	5	FALSE		
GAGGCATCAAAGTGAGCACAG	TTGGA	0.3		chr10:-60854455	-1	4	FALSE		
GCGGCTGCAGACTGAGCCCAC	AGGGA	0.3		chr4:-8109851	-1	4	FALSE		
GCGGCCGCATCGGGAGCCCGG	CGGAA	0.3	NHLRC2	chr10:+113854863	1	4	FALSE		
CAGGCAGCTTAGGGAACCCAG	CTGGG	0.2		chr5:-76447599	-1	5	FALSE		
GCGGCAGGATGAGGAGCCCAG	ACGAG	0.2	KB-176G8.1	chr21:-36486399	-1	4	FALSE		
GAGGCAGCTTTGTGGGCCCAG	CTGGG	0.2		chr17:+81460830	1	4	FALSE		
AAGGCAGCAAAGAGAGCTCAG	CTGAG	0.2		chr6:-98116541	-1	5	FALSE		
TTGGCAGCAAAGGGAGCTCAG	AGGAG	0.2		chrX:+115046270	1	5	FALSE		
TCGGCAGCAGAGAGACCCCAG	CCGAG	0.2		chr15:-40464161	-1	4	FALSE		
GAGGCAGCAAAGCAAGCCCAG	GTGAA	0.2		chr1:+4335197	1	4	FALSE		

Table 2. OFF-target analysis for CCR5-B gRNA.

Sequence	PAM	Score	Gene	Chromosomal location	Strand	Mismatches	On-target	PCR/Sanger sequencing	InDels presence
CTCAGTTTACACCCGATCCAC	TGGGG	**100**	**CCR5**	chr3:+46373928	1	0	TRUE		
CCCAGGTGACACCTGATCCAC	GGGAG	0.5		chr17:-79343644	-1	4	FALSE	YES	**ND**
ACCAGATTACACCTGATCCAA	CGGAA	0.4		chr2:-17940905	-1	5	FALSE	YES	**ND**
ATCTGTTTTCACCAGATCCAA	AGGGA	0.3		chr6:-85491944	-1	5	FALSE	YES	**ND**
CTCATGTTTCACCCGAACCAC	CAGAG	0.3		chrY:+14304163	1	4	FALSE	NO	
GTCAGTGTGAACCCGATCCAG	AGGAG	0.3	SCNN1G	chr16:-23186439	-1	5	FALSE	YES	**ND**
TTCAGCTTACAATCGATACAC	ATGAA	0.2		chr1:-222828548	-1	5	FALSE	YES	**ND**
ATCAGATTTCACCTGCTCCAC	AGGAG	0.2		chr1:-29554118	-1	5	FALSE	YES	**ND**
CTCAGTTTGTACCCGATCCTT	CTGGA	0.1		chr15:+94661446	1	4	FALSE	YES	**ND**
GTCACTTTATACCCGATCAAA	ATGAG	0.1	PTPN3	chr9:-109438163	-1	5	FALSE	YES	**ND**
CTCAGTTTCCACCTGATCCCT	CTGGG	0.1		chr18:-26405641	-1	4	FALSE	YES	**ND**
ACCAGTTTAGATCCAATCCAC	TAGGA	0.1		chr4:+163626400	1	5	FALSE	YES	**ND**
CTCAGATTCCACCCCATCCCC	ACGGG	0.1	RP11-114H24.7	chr15:-77921905	-1	4	FALSE		
ATCAATTTACCCCCAATCCCC	TAGAA	0.1		chr20:+19941694	1	5	FALSE		
TTCAGGTGACACCAGAGCCAC	CAGGG	0.1		chr1:-30508631	-1	5	FALSE		
CTCAGTTTTCACCTGATCAAG	TGGAA	0.1		chr10:-117668370	-1	4	FALSE		
GTCCGTTTACAACCAAGCCAC	AGGAA	0		chr2:-122592489	-1	5	FALSE		
CTCAGTTTACACCTCAACCAC	TGGAA	0		chr11:+57529052	1	3	FALSE		
CTCAGTCTACATCCTTTCCAC	TAGGA	0		chr1:+188857367	1	4	FALSE		
CTCAGTTTCCACCCTGTCCCC	GGGAA	0		chr9:+124323708	1	4	FALSE		
CTCAGTTTAAACCCTTTCCAA	ATGAG	0		chr3:+62969850	1	4	FALSE		
CTCAGTTTCCACCCTAGCTAC	CTGGG	0		chr4:+15608391	1	4	FALSE		

Summary of the OFF-target analysis for gRNAs targeting the human *CCR5* gene. The Benchling CRISPR guides designer tool (https://www.benchling.com) was used to screen the sequence of the human *CCR5* (NCBI: NG_012637) gene for possible gRNA protospacer regions followed by saCas9 specific NNGRR(N) PAM. Next, a pair of gRNAs binding to the coding sequence of the *CCR5* gene (CCR5-A and CCR5-B) was chosen based on the highest on target cleavage score and the lowest nominated off-target cleavage score (strictly at least 4-5 mismatches compared with the target sequence thus making unwarranted cleavage at these sites highly unlikely). The sequences of ON-target (in CCR5 gene, top row) and nominated OFF-target sites in the human genome (with mismatched nucleotides compared to target sequence marked in red), corresponding PAM sequences, predicted cleavage efficiency scores, chromosomal locations, and the number of mismatches is shown for CCR5-A (**Table 1.**) and CCR5-B (**Table 2.**) gRNAs. Additionally, the lack of unintended CRISPR editing in the top ten of nominated OFF-target sites for each gRNA was experimentally verified by PCR-genotyping/Sanger sequencing using as a template genomic DNA from two CCR5k/o clones (C14 and C18, carrying complete

biallelic □767bp CCR5-A to CCR5-B excision of *CCR5* gene) and two control clones (Ctrl1 and Ctrl2) generated for this study in TZM-bl cell line. As indicated in the last column, no Indel mutations were detected at the investigated genomic locations across all the cellular clones tested, proving a lack of OFF-target activity of CCR5-A and CCR5-B gRNAs. ND – not detected.

A.

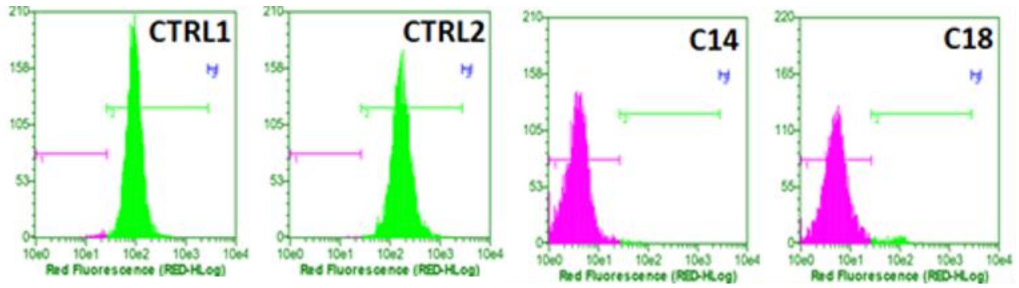

B.

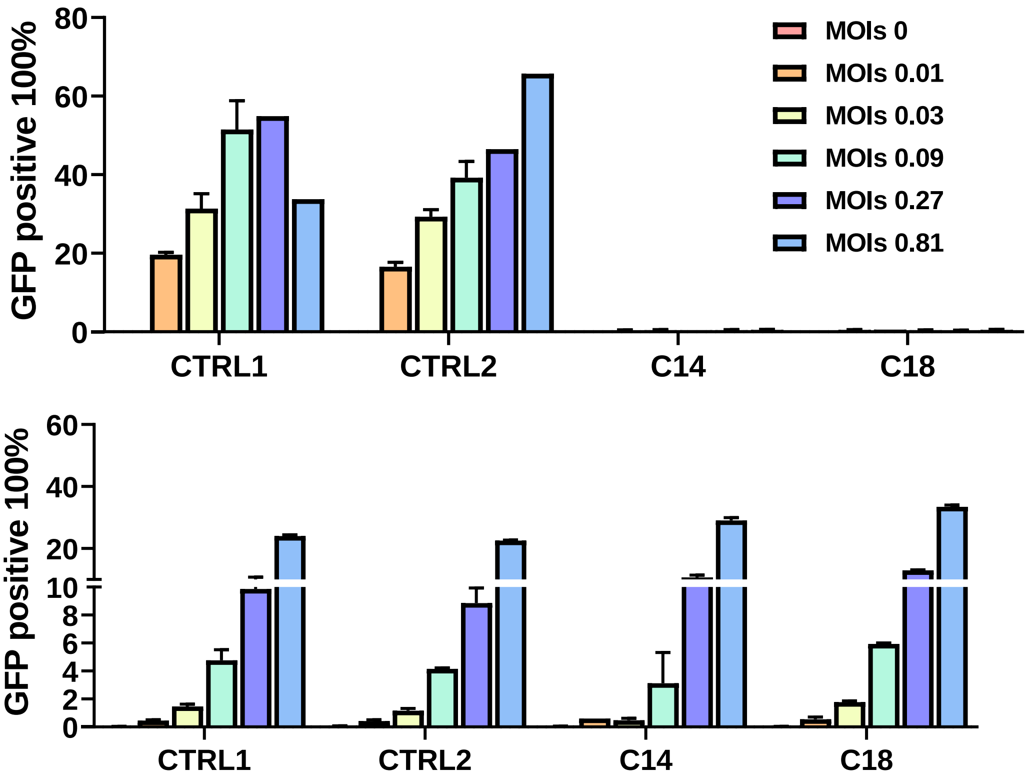

Supporting Data Fig. 1. Knockout of CCR5 gene expression protects cells from infection by CCR5-tropic HIV-1. (A) Immunolabelling/flow cytometry analysis of CCR5 antigen expression in control (CTRL1 and CTRL2) and CCR5 knockout (C14 and C18) TZM-bl cells. **(B)** Control and CCR5 knockout cells were infected with CCR5-tropic HIV-1 NL4-3-BAL-GFP (top panel) or pantropic HIV-1 NL4-3-BAL-GFP/VSV-g (bottom panel) at different multiplicities of infection (MOIs, from 0.01 to 1). 48h later, GFP expression was measured by flow cytometry on paraformaldehyde-fixed cells. As expected, CCR5 knockout cells (C14 and C18) were resistant to infection with CCR5-tropic but not to infection with pan-tropic, VSV-g pseudotyped HIV-1.

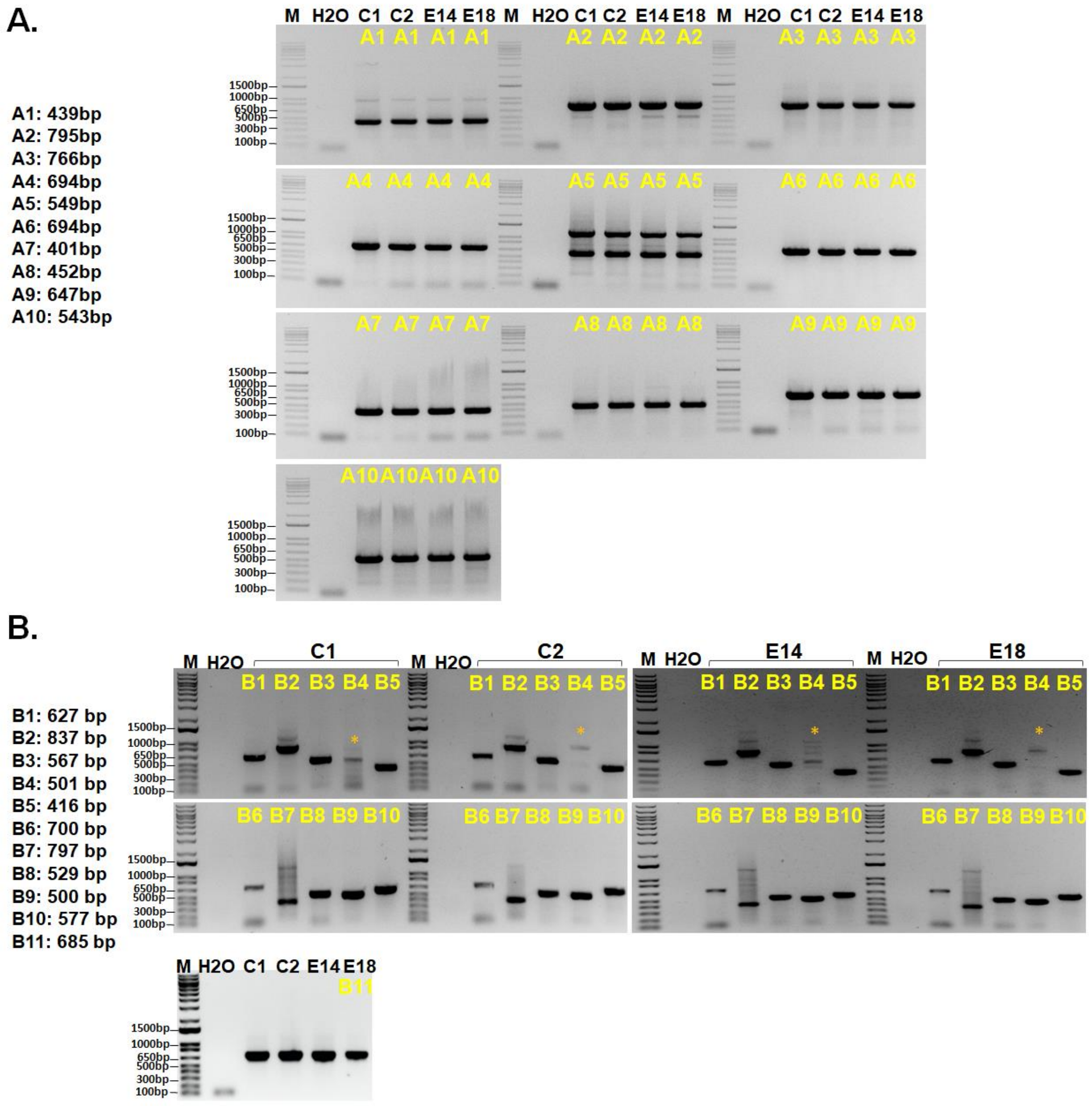

Supporting Data Fig. 2. PCR-genotyping of top ten *in silico* nominated off-target sites for gRNAs CCR5-A and CCR5-B in Wild Type and CCR5-knockout TZM-bl single cell clones. Agarose gel analysis of PCR genotyping of top ten bioinformatically nominated off-target sites in the human genome for CCR5-A (**panel A,** A1-A10) and CCR5-B (**panel B,** B1-B11) gRNAs. Genomic DNAs from two control (C1 and C2) and two CCR5-knockout (E14 and E18, carrying complete biallelic ∆767bp CCR5-A to CCR5-B excision of *CCR5* gene) TZM-bl single cell clones generated for this were used as PCR templates. Clone/locus-specific amplicons were purified from the gels and sequenced and results are summarized in the last column of **Supporting**

Tables 1 and 2. No Indel mutations were detected at the investigated genomic locations across all the cellular clones tested, proving a lack of off-target activity of CCR5-A and CCR5-B gRNAs. Predicted amplicon sizes are shown on the left. The lines in agarose gels corresponding to specific amplicons are labeled in yellow. For gRNA CCR5-B the fourth top nominated off-target site locus (B4) is located on chromosome Y so yielded no specific amplification (indicated as yellow asterisks) from genomic DNA from TZM-bl cells which were derived from a female donor. Thus, the eleventh top nominated off-target site was added to the panel for CCR5-B gRNA.

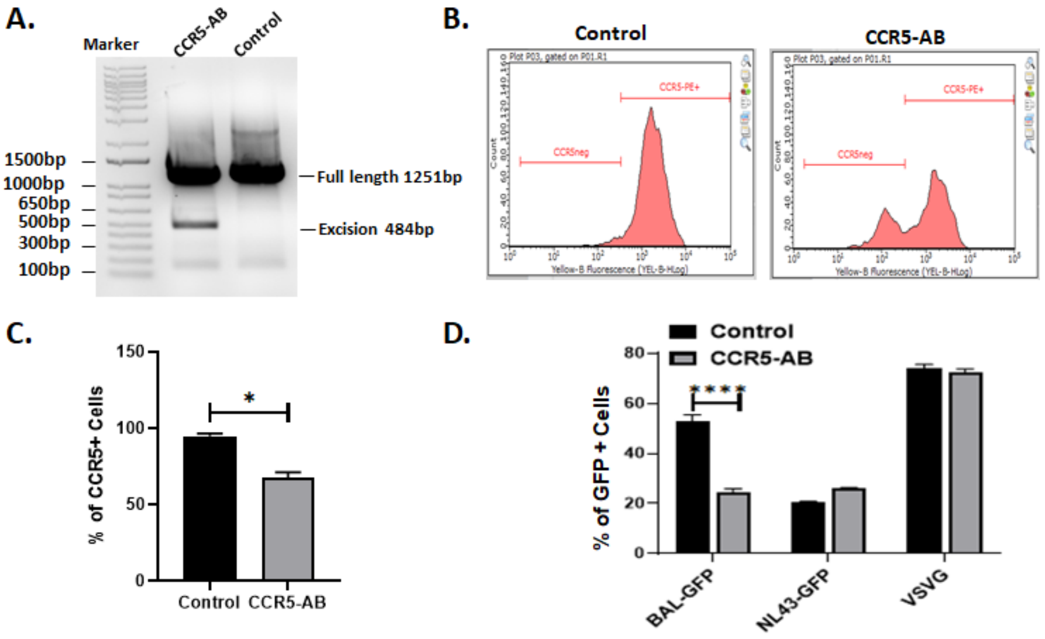

Supporting Data Fig. 3. Downregulation of CCR5 in CD4+ T cells protects against HIV-1 infection by CCR5-tropic HIV-1. (A) Agarose gel electrophoretic analysis of PCR genotyping of CRISPR-Cas9-mediated excision of CCR5 gene. Genomic DNAs from CRISPR-CCR5-treated and control HutR5 cells were used as PCR templates. (**B**) Representative flow cytometry histograms for control and CRISPR-CCR5-AB treated cells. (**C**) Immunolabelling/flow cytometry analysis of CCR5 antigen expression in control Cas9-only or Cas9/CCR5-AB treated HutR5 cells. (**D**) Control (black bars) and CRISPR-CCR5-AB treated (grey bars) HutR5 cells were infected with CCR5-tropic HIV-$_{\text{1NL4-3-Bal-GFP}}$, CXCR4-tropic HIV-1$_{\text{NL4-3-GFP-P2A-Nef}}$ or pan-tropic VSV-g-pseudotyped HIV-1$_{\text{NL4-3-GFP-P2A-Nef}}$. 48h later, GFP expression was measured by flow cytometry on paraformaldehyde-fixed cells. As expected, CRISPR-CCR5-AB treated cells show resistance to infection with CCR5-tropic but not to infection with CXCR4-tropic HIV-1.

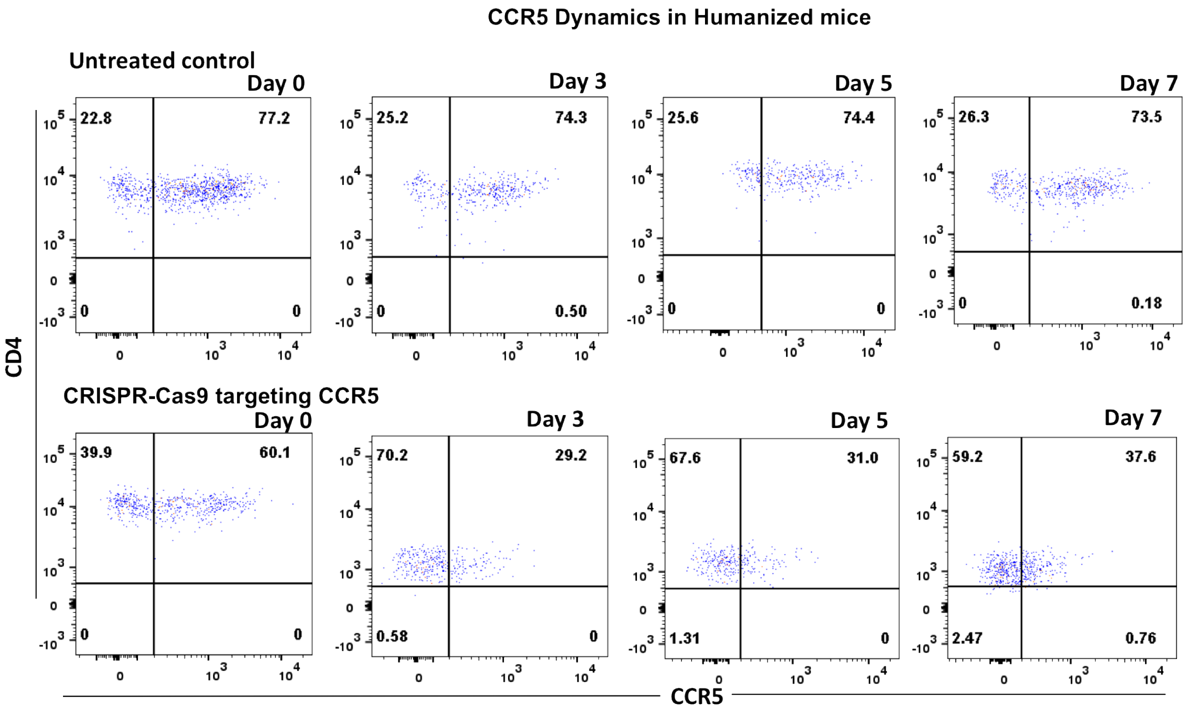

Supporting Data Fig. 4. CCR5 expression in immunocytes of humanized mice. CCR5 expression from CD3+CD4+ T cells from a representative hu-mouse injected "treated" with AAV6 CCR5 CRISPR-Cas9 excision and one control. Mice were bled prior to and 3, 5 and 7 days after injection. CCR5 expression was diminished for up to 7 days in the AAV6 CCR5 CRISPR-Cas9 treated mice compared to untreated controls. CCR5 CRISPR-Cas9 was administered to a group of 5 mice through AAV6 delivery by a tail vein intravenous injection. A replicate group of 5 untreated hu-mice served as controls.

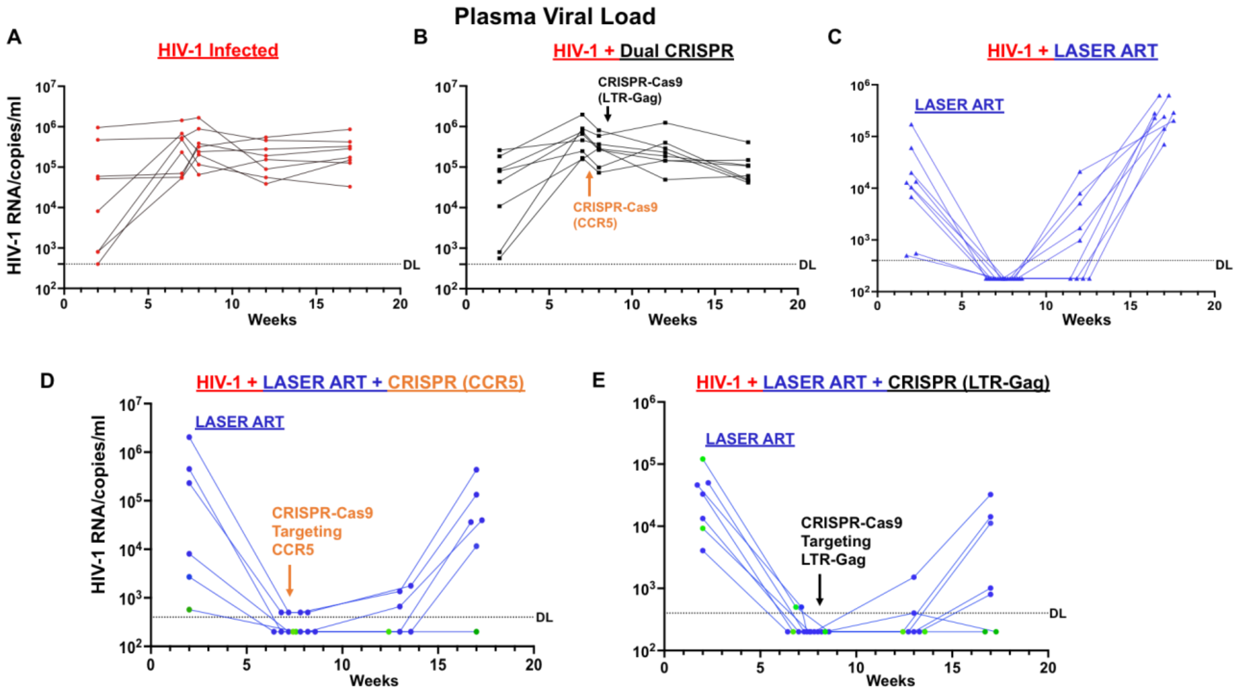

Supporting Data Fig. 5. Plasma viral load of individual Humanized mice of untreated, ART and single CRISPR treatments. (**A**) Plasma HIV-1 RNA copies in untreated control hu-mice (n=8, shown in red), **(B)** infected and ART and AAV6-CRISPR-Cas9 CCR5 and AAV9 CRISPR-Cas9 HIV-1 LTR Gag genes (n=8, shown in black line) and **(C)** HIV-1_{ADA} infected LASER ART treated (n=9, shown in blue). Each of the treatments followed two-weeks of virus infection of hu-mice. Viral rebound was observed at study end in all animals after ART withdrawal. The dots represent individual mice in each group throughout the time course. (**D**) Plasma viral load of individual animals (n=6) treated with LASER ART and AAV6 CCR5 CRISPR-Cas9. Five of 6 hu-mice showed viral rebound at 17 weeks following viral infection. (**E**) Plasma viral load of individual animals (n=7) treated with LASER ART and CRISPR-Cas9 targeting HIV-1 LTR Gag. Five of 7 animals showed viral rebound. Plasma viral load of the individual animal groups was assayed at 2, 7, 8, 13 and 17 weeks after viral infection. HIV-1 RNA was determined by the COBAS Ampliprep-Taqman-48 V 2.0 assay. The sensitivity of detection for the mice were at 200 copies/ml after adjustments for plasma dilution.

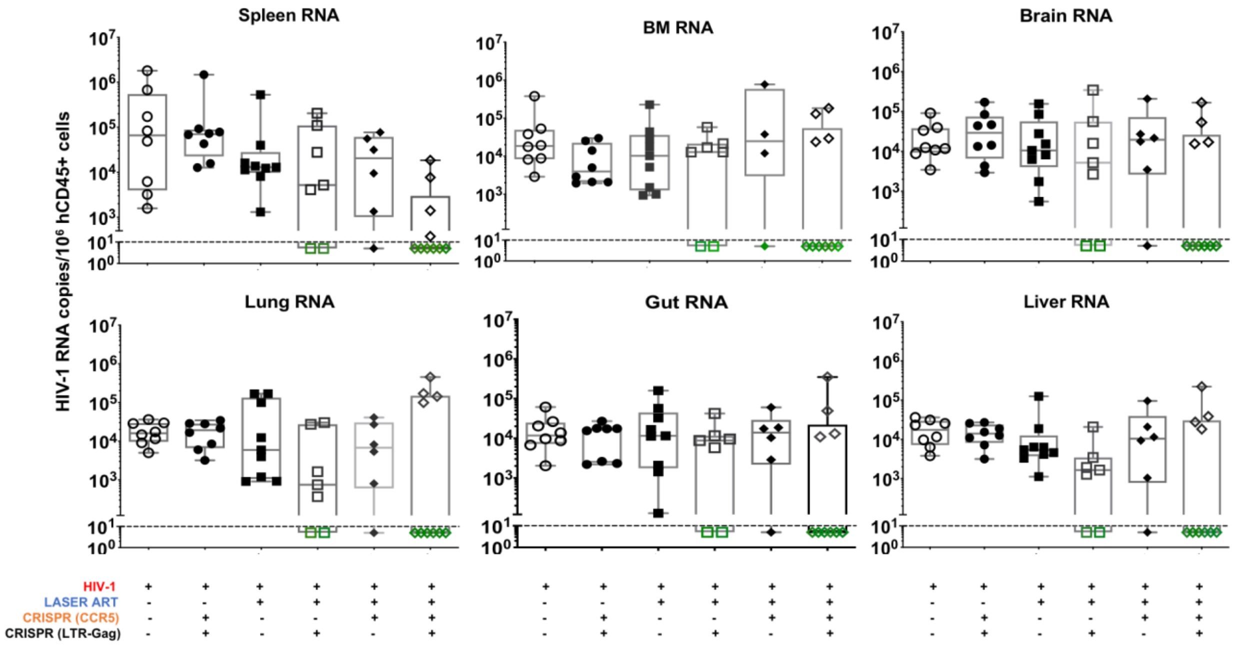

Supporting Data Fig. 6. Viral RNA in HIV-1 infected and LASER ART and CRISPR treated hu-mice tissues. HIV-1 RNA levels were measured in the gag region of HIV-1 using ultrasensitive semi-nested real-time qPCR assays. These tests were performed in spleen, lung, gut, bone marrow, brain, and liver from each of the treatment groups. The data represent the following: HIV-1 infected (n=8), HIV-1 infected and dual CRISPR-Cas9 treated (n=8), HIV-1 infected and LASER ART treated (n=9), HIV-1 infected and LASER ART treated and CRISPR CCR5 (n=6), HIV-1 infected LASER ART treated and CRISPR LTR Gag (n=7) and HIV-1 infected and LASER ART and CRISPR CCR5 and LTR Gag treated mice (n=10). One of six animals from LASER ART and CRISPR CCR5 group, two out of seven mice from ART and CRISPR HIV-1 LTR Gag group and six out of ten animals from LASER ART and CRISPR CCR5 and HIV-1 LTR Gag group demonstrated viral elimination. The data are shown in green open boxes. For these hu-mice viral amplification assay failed to demonstrate viral nucleic acid. The detection limit of the assay is 10 copies of viral RNA. The data are expressed as total HIV-1 RNA copies/10^6 human CD45+ cells. The data represent mean ± SEM for each group.

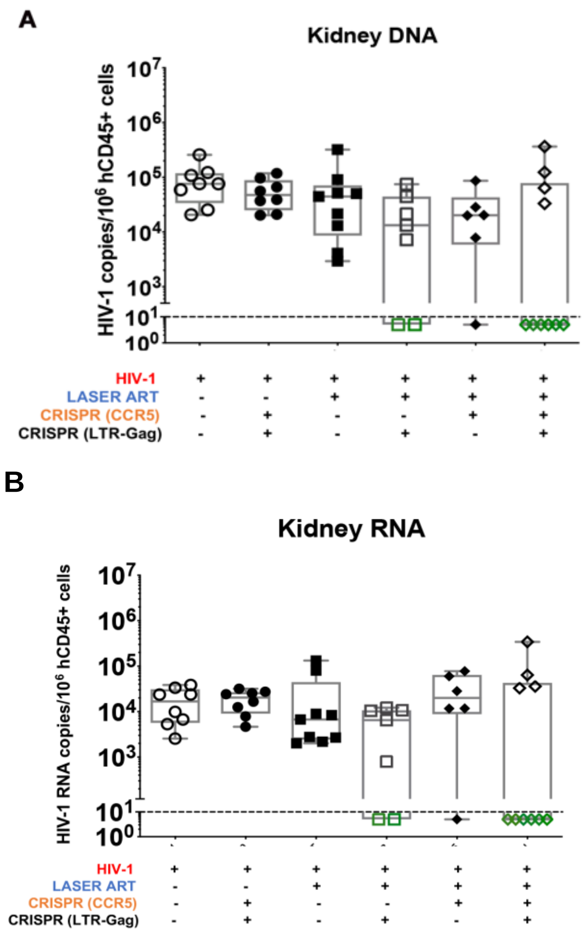

Supporting Data Fig. 7. Viral DNA and RNA in HIV-1 infected, LASER ART and CRISPR treated hu-mice. HIV-1 DNA (**A**) and RNA (**B**) analyses in the gag region using ultrasensitive semi-nested real-time qPCR assays from kidney tissues. The data sets are shown from different treatment groups employed. Each plate represents the six individual groups. These include HIV-1 infected (n=8), HIV-1 infected and CRISPR treated (n=8), HIV-1 infected and LASER ART treated (n=9), HIV-1 infected, LASER ART treated and CRISPR targeting CCR5 (n=6), HIV-1 infected, LASER ART treated and CRISPR HIV-1 LTR-Gag (n=7) and HIV-1 infected, LASER ART treated and CCR5 and HIV-1 LTR Gag CRISPR mice (n=10). One out of six animals from LASER ART and CRISPR CCR5 and two out of seven animals from LASER ART and CRISPR HIV-1 LTR Gag group and six out of ten animals in the LASER ART and CRISPR CCR5 and HIV-1 LTR Gag groups failed to demonstrate virus. These are shown in green open boxes and showed no viral amplification. The detection limit of the assay is 10 copies of viral DNA/RNA. The data are expressed as total HIV-1 DNA/RNA copies/10^6 human CD45+ cells. The data represent mean ± SEM for each group.

RNAscope (Spleen)

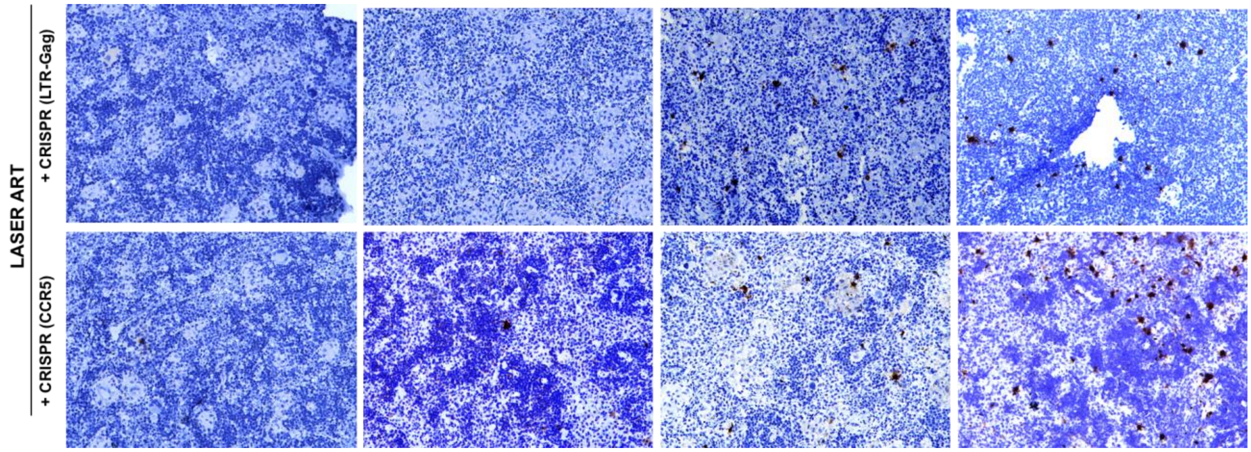

Supporting Data Fig. 8. RNAscope assays. Representative results from RNAscope assay revealed the detection of single or clusters of brown dots corresponding to HIV-1 RNA in 5 μm-thick spleen sections of infected animals receiving LASER ART and CRISPR targeting CCR5 and HIV-1 LTR Gag. Top panel shows HIV-1 infected animals treated with ART and AAV9 mediated CRISPR targeting HIV-1 LTR Gag. Here viral rebound was demonstrated in 5/7 animals after therapeutic cessation. Bottom panel shows hu-mice infected with HIV-1, treated with LASER ART and CRISPR CCR5; 1/6 had no detectable HIV-RNA, in this group. The figures are representative tissue sections taken from each of the animal groups. In these assays, we used the antisense V-HIV1-Clade-B targeting 854–8291 bp of HIV-1 as the probe. Images were captured at 20x magnification. Human peptidyl Isomerase B was used as a positive control for the analyzed tissues.

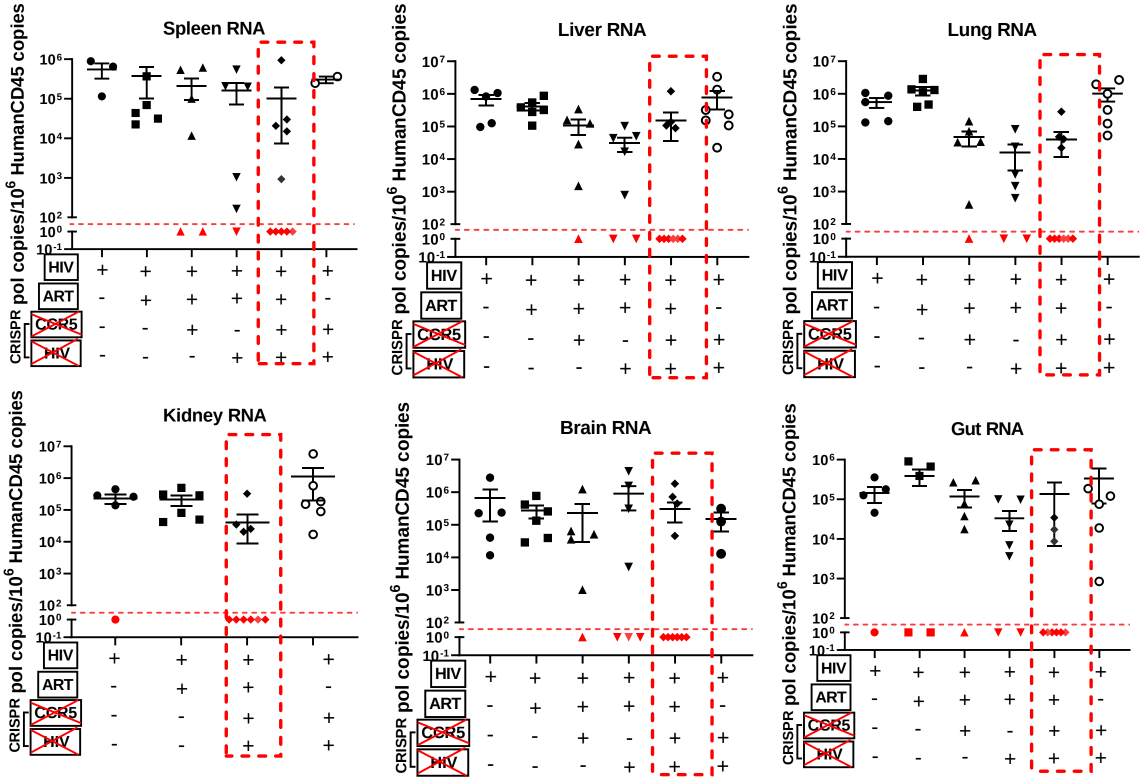

Supporting Data Fig. 9. Viral RNA in HIV-1 infected and CRISPR treated hu-mice tissues. HIV-1 RNA in the viral polymerase region using the ddPCR assay from spleen, lung, gut, brain, liver, and kidney tissues from described treatment groups. The data represent each of the six groups HIV-1 infected (n=5), HIV-1 infected and CRISPR CCR5-HIV-1 treated (n=5), HIV-1 infected and LASER ART treated (n=6), HIV-1 infected and LASER ART treated and CRISPR CCR5 (n=6), HIV-1 infected, LASER ART and CRISPR HIV-1 LTR Gag (n=7) treated, and HIV 1 infected and LASER ART and CRISPR CCR5 and HIV-1 LTR Gag treated mice (n=10). The detection limit of the assay is 2 copies of viral RNA. The data are expressed as total HIV-1 RNA copies/106 human CD45 copies. The data represent mean ± SEM for each group.

A

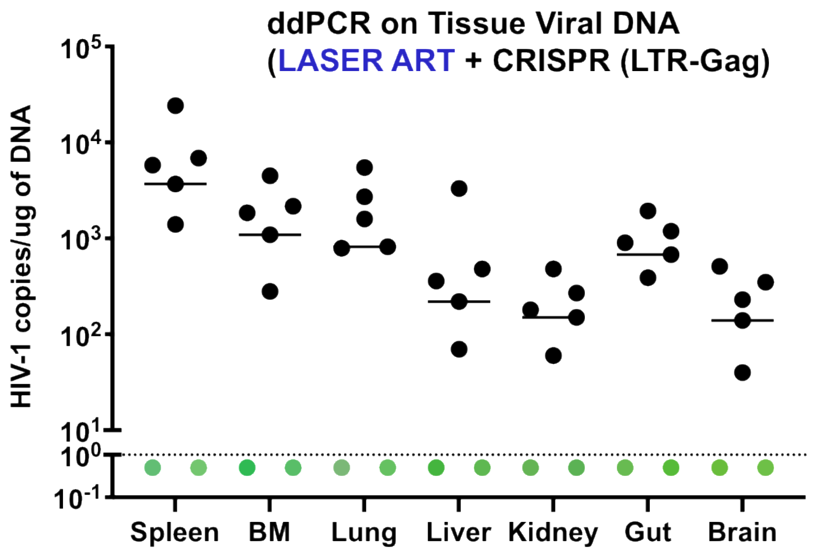

B

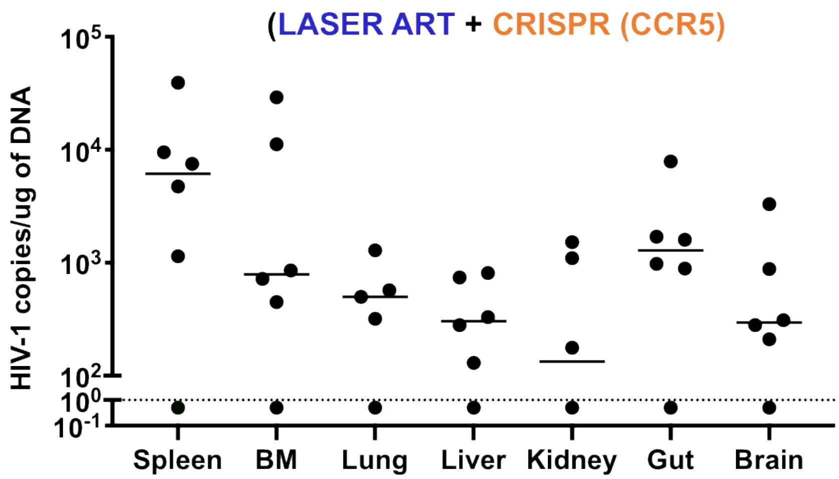

Supporting Data Fig. 10. Viral DNA in HIV-1 infected, LASER ART and CRISPR treated hu-mice tissues. HIV-1 DNA analyses in the HIV-1 Gag region using the ddPCR assay in spleen, lung, gut, brain, liver, and kidney tissues from different treatment groups as described in Extended Data Fig. 3. The data represent each of the two groups. (**A**) HIV-1 infected LASER ART and CRISPR LTR Gag treated (n=7) and **(B)** is HIV-1 infected, LASER ART and CRISPR CCR5 treated (n=6) (**B**). The detection limit of the assay is 2 copies of viral DNA. One/six animals from LASER ART+ CRISPR (CCR5) group and 2/7 animals from LASER ART + CRISPR (LTR-Gag) group showed no amplification of virus from all the tissues analyzed. The data are expressed as total HIV-1 DNA copies/10^6 human CD45+ cells.

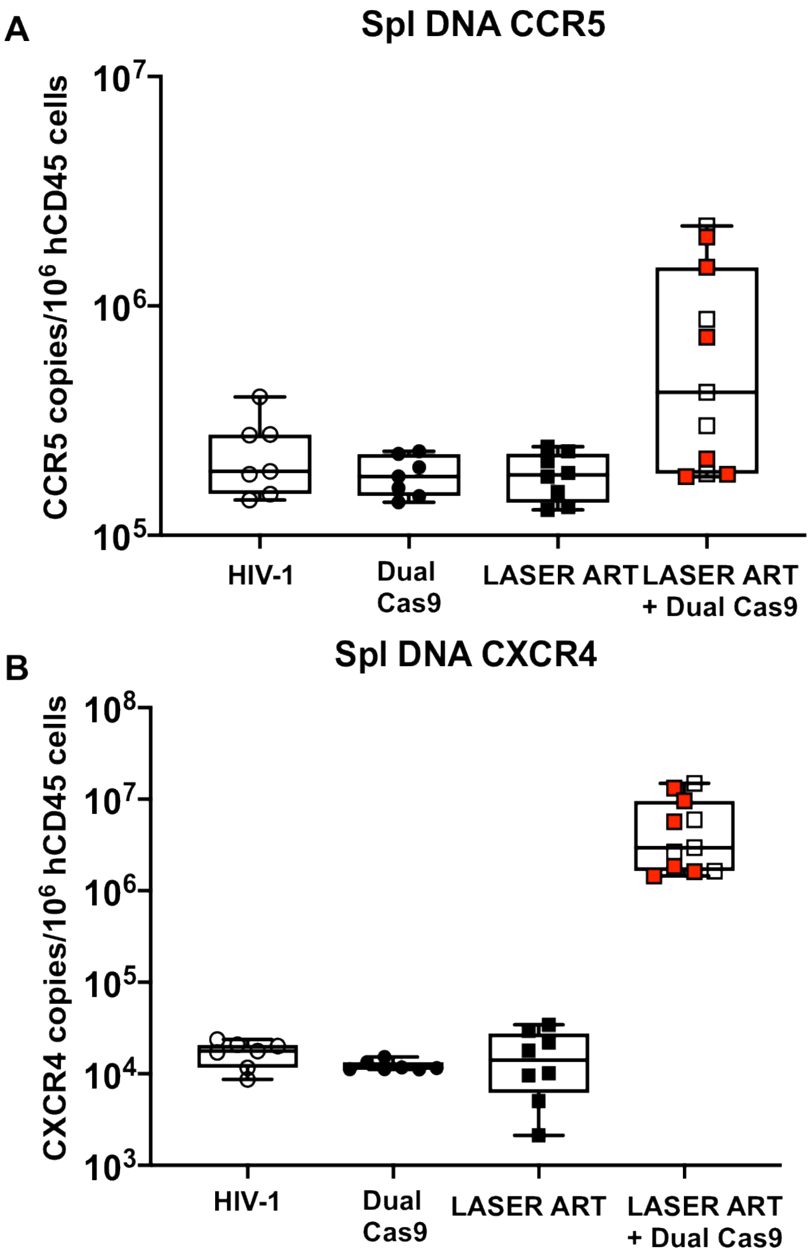

Supporting Data Fig. 11. CCR5 and CXCR4 expression in spleen. DNA analyses of CCR5 (**A**) and CXCR4 (**B**) expression using ultrasensitive semi-nested real-time qPCR assay from spleen tissues of individual animals from four treatment groups at the study end. This confirms the presence of abundant CCR5 and CXCR4 expression. The red filled boxes in the dual treatment group are the animals with LASER ART and dual CRISPR treated with no HIV-1 amplification from previous figures and had the CCR5 expression restored. The data are expressed as total DNA copies/10^6 human CD45+ cells. The data represent mean ± SEM for each group.

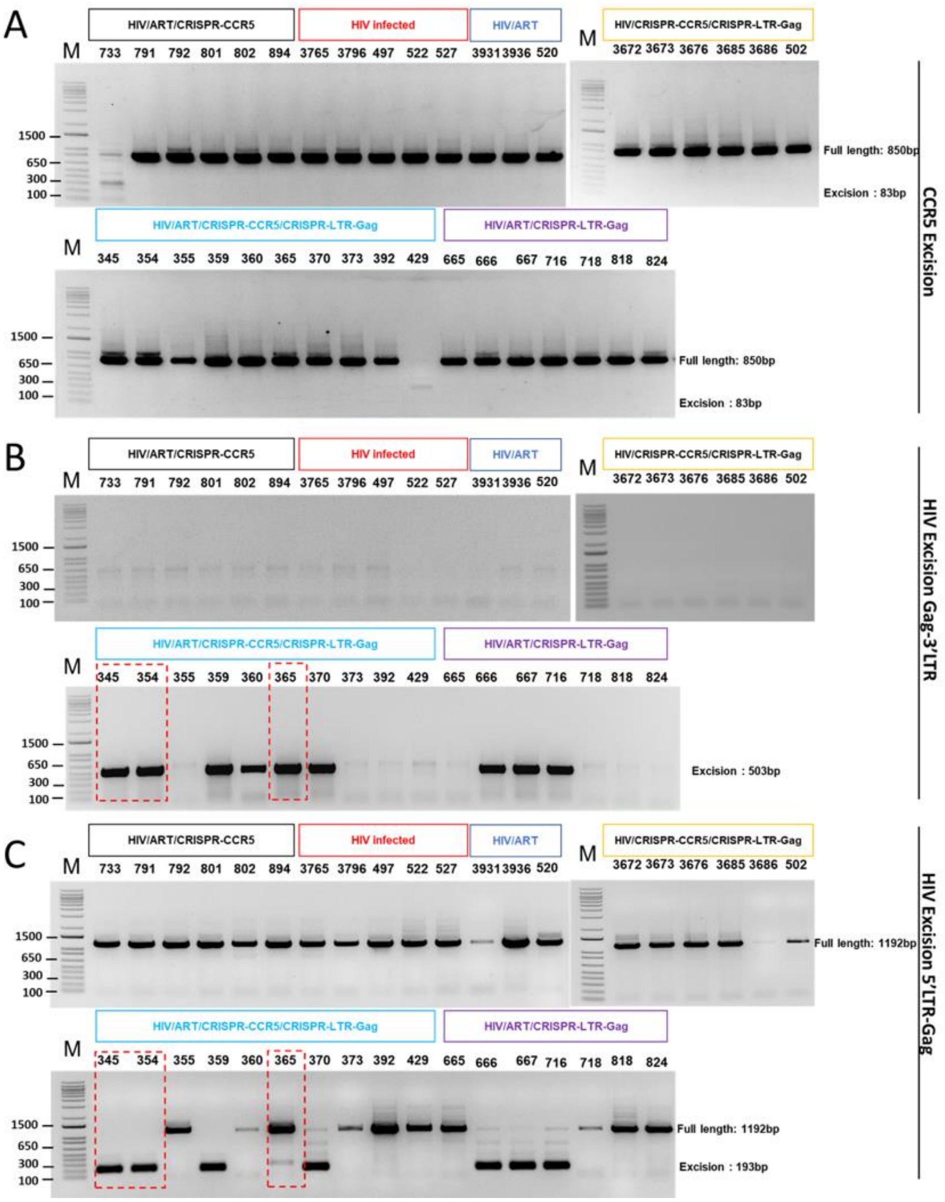

Supporting Data Fig. 12. CCR5 and HIV-1 excision in brains of infected and treated hu-mice. CCR5 and HIV-1 excision study was performed on brain post-LASER ART with or without a single or dual CRISPR-Cas9 injection in infected humanized mice. Total DNA from brain of all untreated/treated animals is used for PCR genotyping with primer sets derived from the 5'LTR, 3'LTR, and the HIV-1 gag and CCR5 gene. (**A**) shows excision of CCR5 DNA in HIV-1 infected humanized mice, as the CCR5 expression came back to normal, we didn't observe the excised band, except in one mouse. (**B**) shows excision of HIV-1 DNA from 3'LTR to Gag by CRISPR-Cas9 in HIV-1 infected humanized mice and (**C**) shows CRISPR-Cas9 excision of HIV-1 Gag to 5'LTR in infected humanized mice.

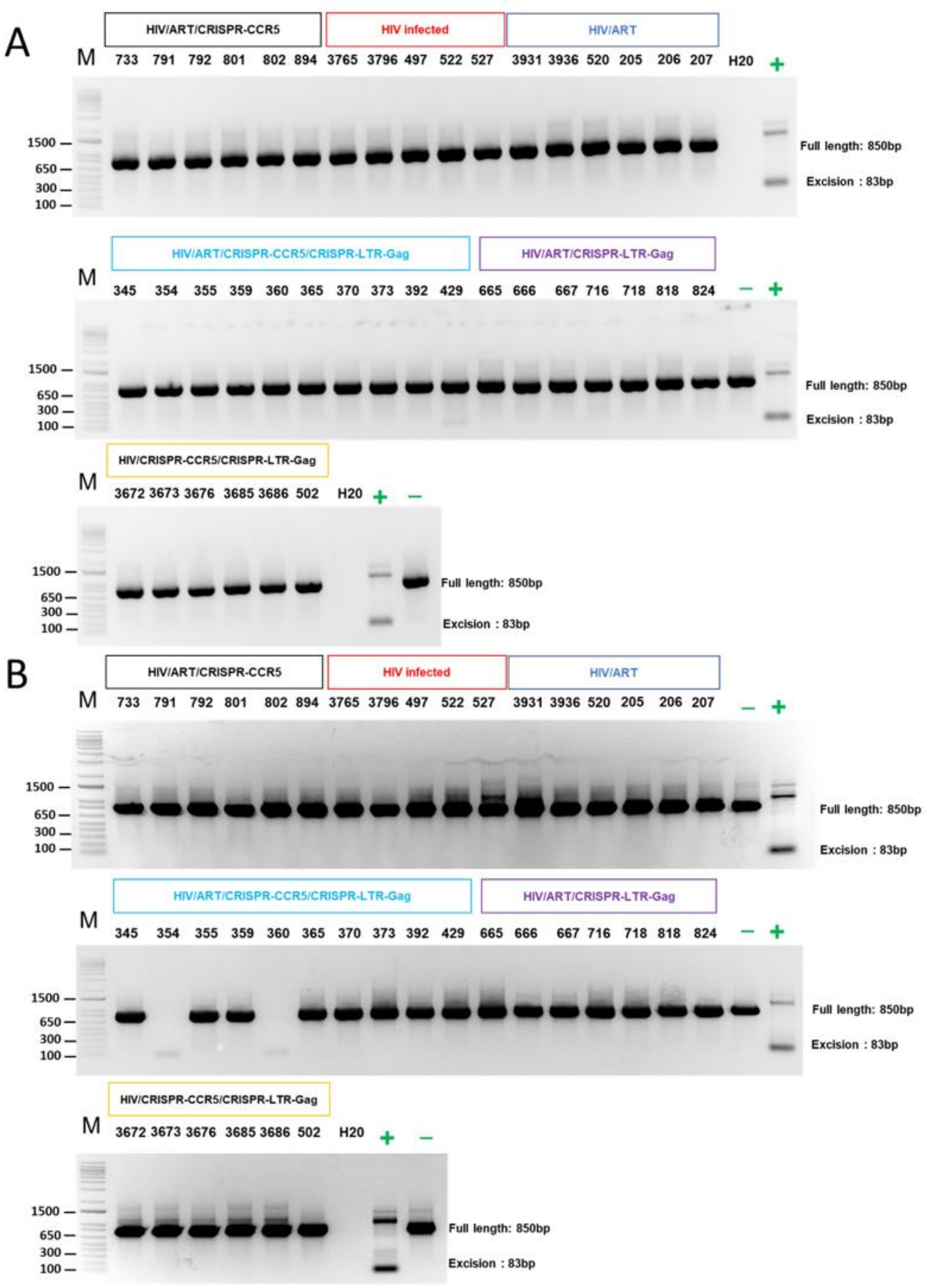

Supporting Data Fig. 13. CCR5 excision in lung and liver of infected and treated humanized mice. CCR5 excision study on lung (**A**) and liver (**B**) tissues post-LASER ART and without or single or dual CRISPR-Cas9 injection in infected humanized mice. Total DNA from lung and livers from all untreated/treated animals were used for PCR genotyping with primer sets for CCR5 gene. Panel shows no excision band of CCR5 DNA in HIV-1 infected humanized mice, as the CCR5 expression came back to normal at study end.

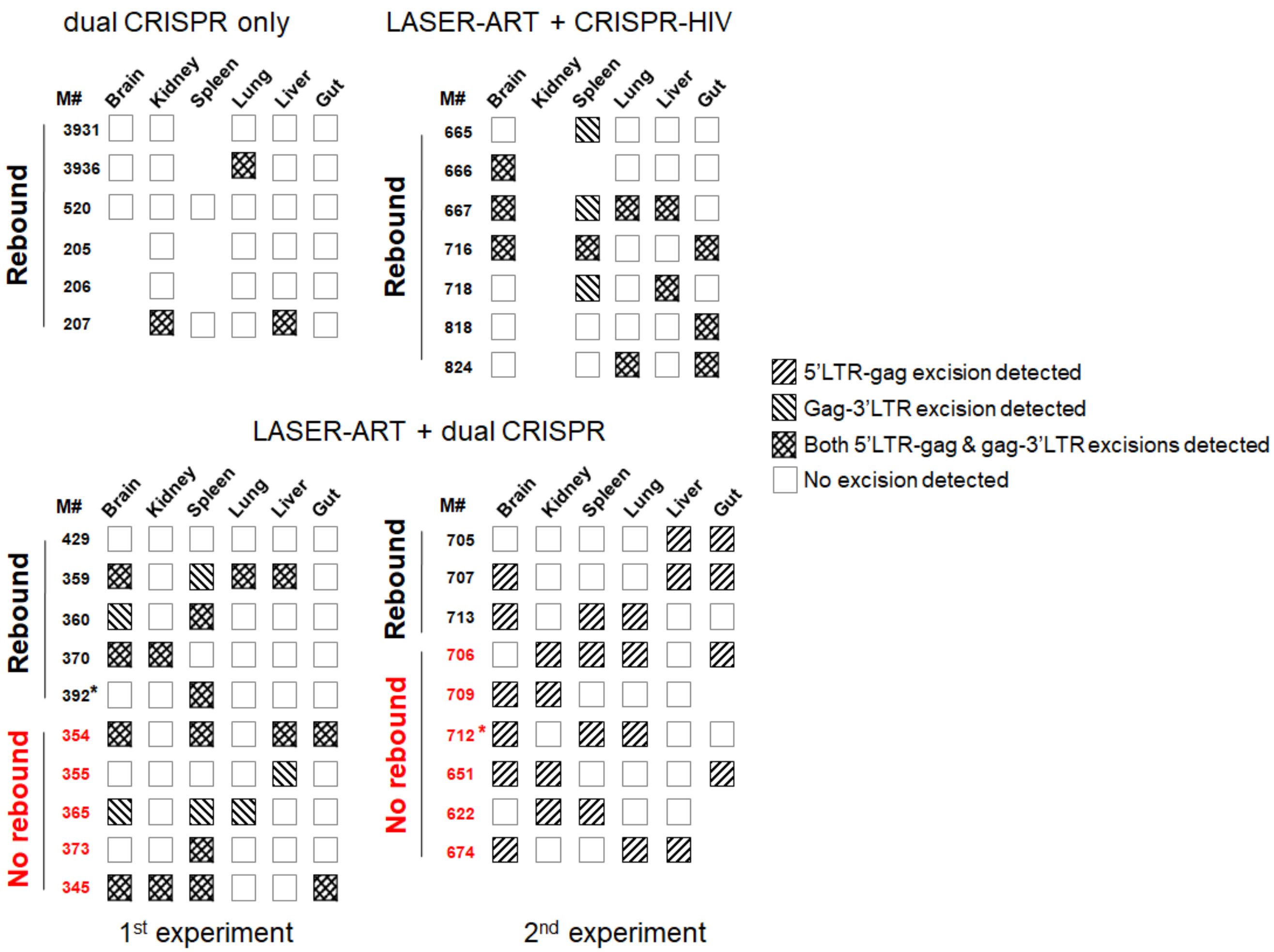

Supporting Data Fig. 14. Summary of HIV-1 excision analysis. The presence of CRISPR-Cas9-mediated excision of proviral sequences in tissues of AAV_9-CRISPR-HIV treated animals were checked by PCR-genotyping followed by verification of detected CRISPR-cleaved/end-joined truncated amplicons by Sanger sequencing. Two CRISPR-Cas9-mediated excision events were investigated: 5'LTR-gag (Δ978bp) and gag-3'LTR (Δ8097bp). Squares represent tested tissue samples. Tissues that were not available for analysis are shown as empty spaces. The animals showing a lack of viral rebound (undetectable plasma viral RNA) are marked red (11 out of 19, =58%). One animal from the first set that received LASER ART and dual CRISPR-Cas9, #392 (marked with a black star), had a very low 400 HIV-1 RNA copies/ml in plasma at 17WPI but no evidence of viral RNA (RNAscope) or DNA (qPCRs and ddPCRs) in the examined tissues. Another animal from the second set of LASER ART and dual CRISPR-Cas9 treated animals, #712 (marked with a red star), had undetectable plasma viral RNA but tested positive for viral DNA in tissues using ddPCR.

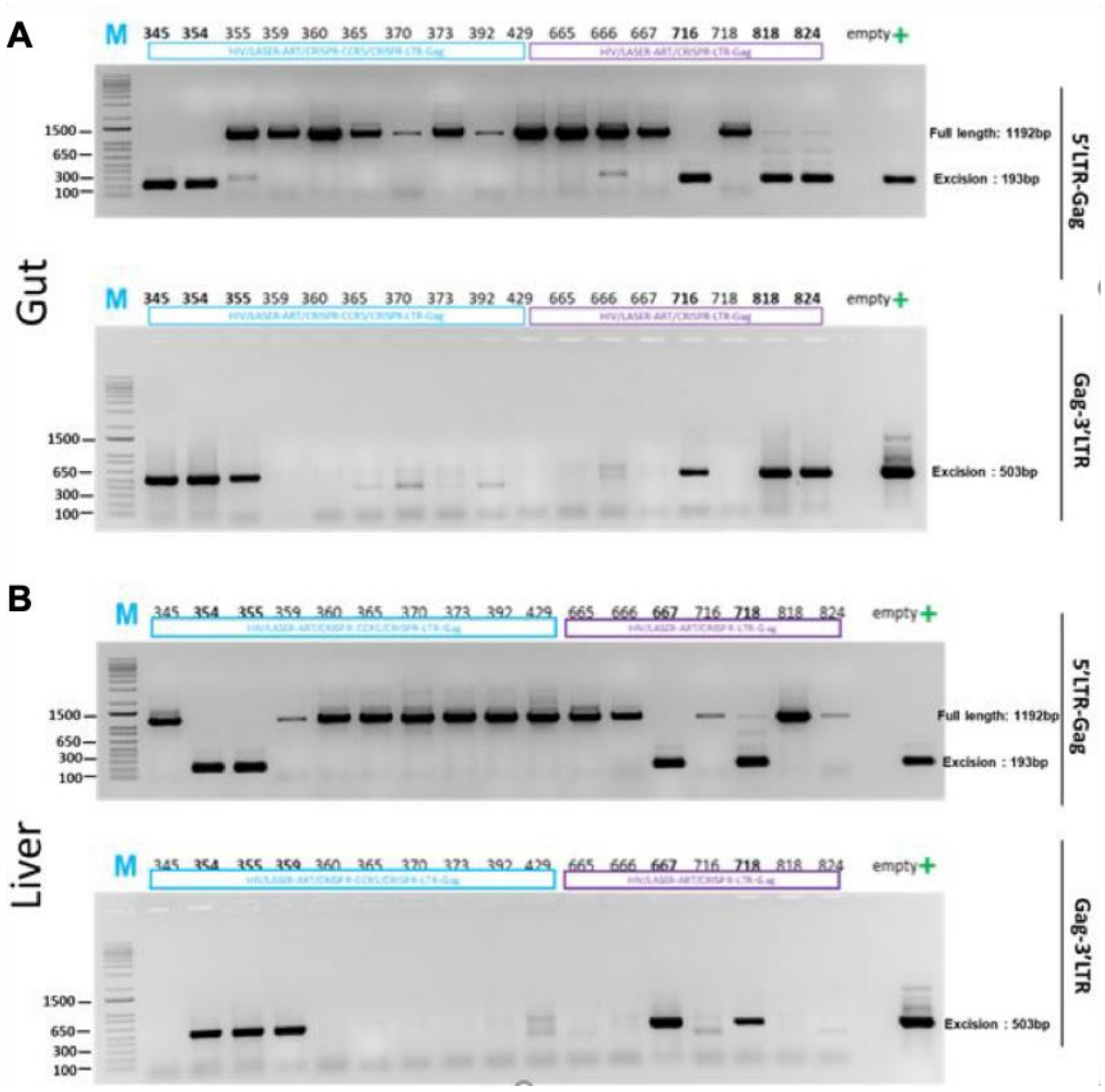

Supporting Data Fig. 15. HIV excision study in gut and liver of infected and treated humanized mice. HIV excision study on gut (**A**) and liver (**B**) tissues from LASER ART and with single or dual CRISPR-Cas9 treatments in infected hu-mice. Total DNA from gut and liver from all single or dual treated animals are used for PCR genotyping with primers sets derived from the 5'LTR, 3'LTR, and the HIV-1 gag gene. The figure shows excision of HIV-1 DNA from 3'LTR to Gag by CRISPR-Cas9 in HIV-1 infected humanized mice and CRISPR-Cas9 excision of HIV-1 Gag to 5'LTR in infected humanized mice. The excised band for 5'LTR-gag is 193 bp and for gag-3'LTR is 503 bp as highlighted.

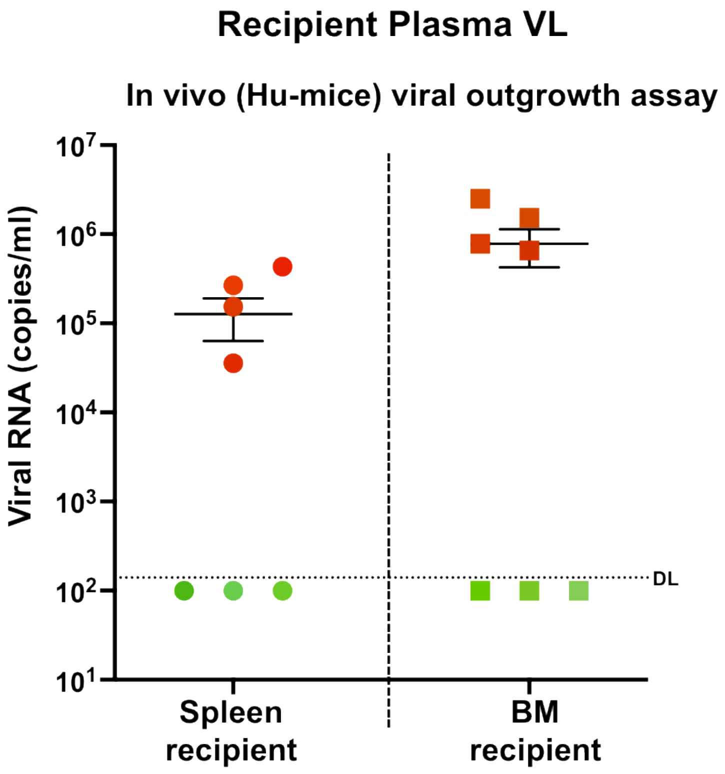

Supporting Data Fig. 16. Viral outgrowth assay to detect replication-competent virus in dual treated humanized mice. The assay was performed by adoptive transfer of splenocytes and BM cells (~ 8-10 x 10^6 cells/mice/tissues) from LASER ART and dual CRISPR-Cas9 treated animals (7 humanized mice) to uninfected recipient CD34 NSG-humanized mice (14 mice, 7 received splenocytes and other 7 received BM cells). Cells isolated from same virally suppressed animals failed to show viral recovery after five weeks of examination by plasma viral RNA measurements as shown in green circles and boxes and used as the definition for viral eradication. We did not get sufficient cells from the other two dual treated hu-mice (622 and 674) to perform adoptive transfer.

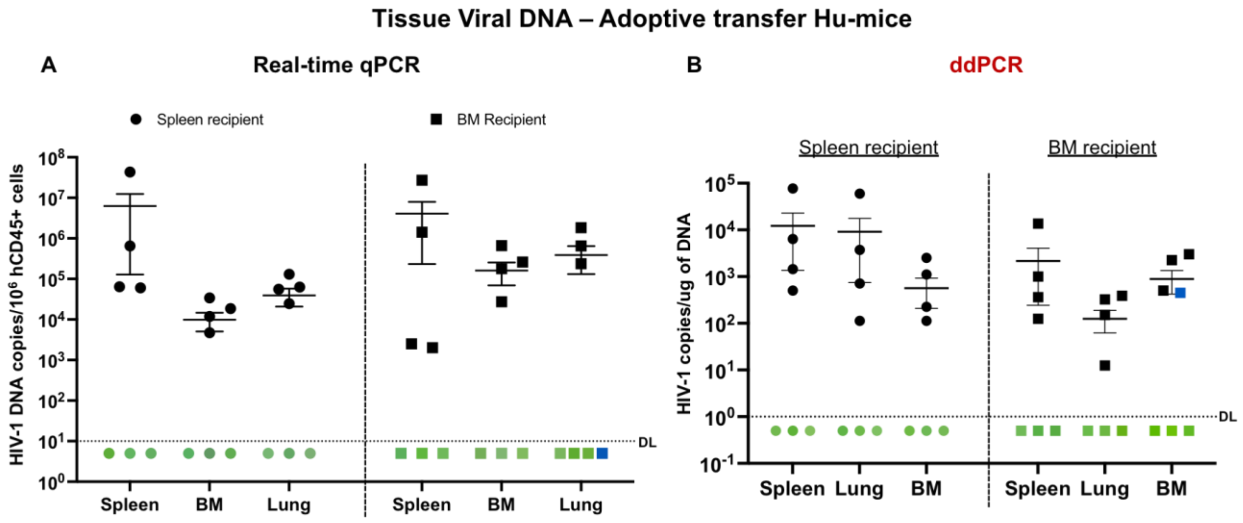

Supporting Data Fig. 17. Detection of HIV-1 DNA in tissues in adoptively transferred humanized mice. Splenocytes and bone marrow (BM) cells were isolated from HIV-1 infected mice with prior LASER ART and dual CRISPR treatment and used for adoptive transfers into new CD34+ NSG-humanized mice. The intent was to perform cross disciplinary viral amplification from known infectious cell reservoirs. (**A**) HIV-1 DNA analyses using semi-nested real-time qPCR assays from spleen, bone marrow and lung tissues of adoptively transferred humanized mice. The data are expressed as total HIV-1 DNA copies/10^6 human CD45+ cells. Three animals shown by green circles and squares below dotted line), showed no viral recovery. We couldn't get enough cells from another 2 mice for adoptive transfer. The above data were further confirmed using the ddPCR assay (**B**), where the adoptively transferred recipient animals failed to demonstrate HIV-1 products indicating complete viral elimination. Virus was recovered from all rebound mouse tissues. The data are expressed as total HIV-1 DNA copies/microgram of DNA used for ddPCR assay after normalization to human cells. The blue color dot mice were negative in semi-nested real-time qPCR but was found to be positive in ddPCR, highlighting the importance of using a sensitive assay (detection limit of 1-2 copies) for viral detection. The detection limit for real-time qPCR is 10 copies and is 1 copy for ddPCR. The data represent mean ± SEM for each group.

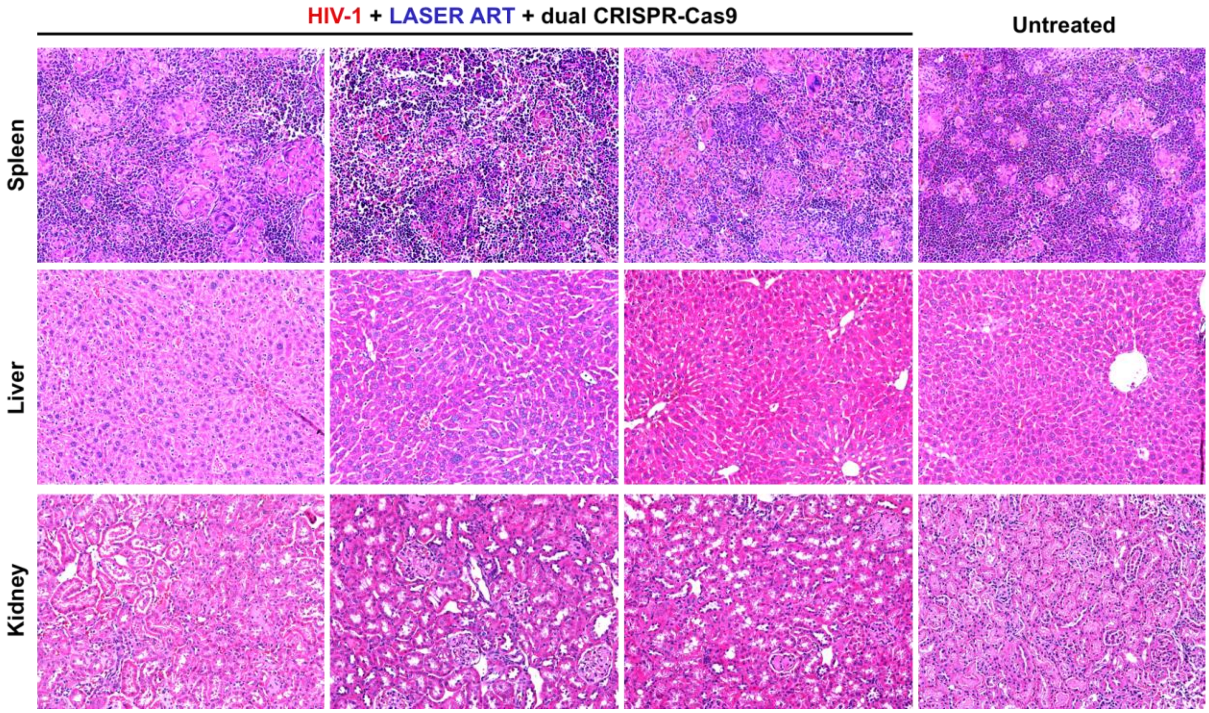

Supporting Data Fig. 18. Liver, kidney, and spleen tissue histology in treated hu-mice. Hematoxylin and eosin staining of representative sections from liver, kidney, and spleen tissues in HIV-1_{ADA}-infected, LASER ART treated and dual CRISPR injected humanized mice at the endpoint of the study. We did not observe any tissue pathology in LASER ART alone or dual treatment groups as compared to untreated controls. The images were captured at 20-x magnification.

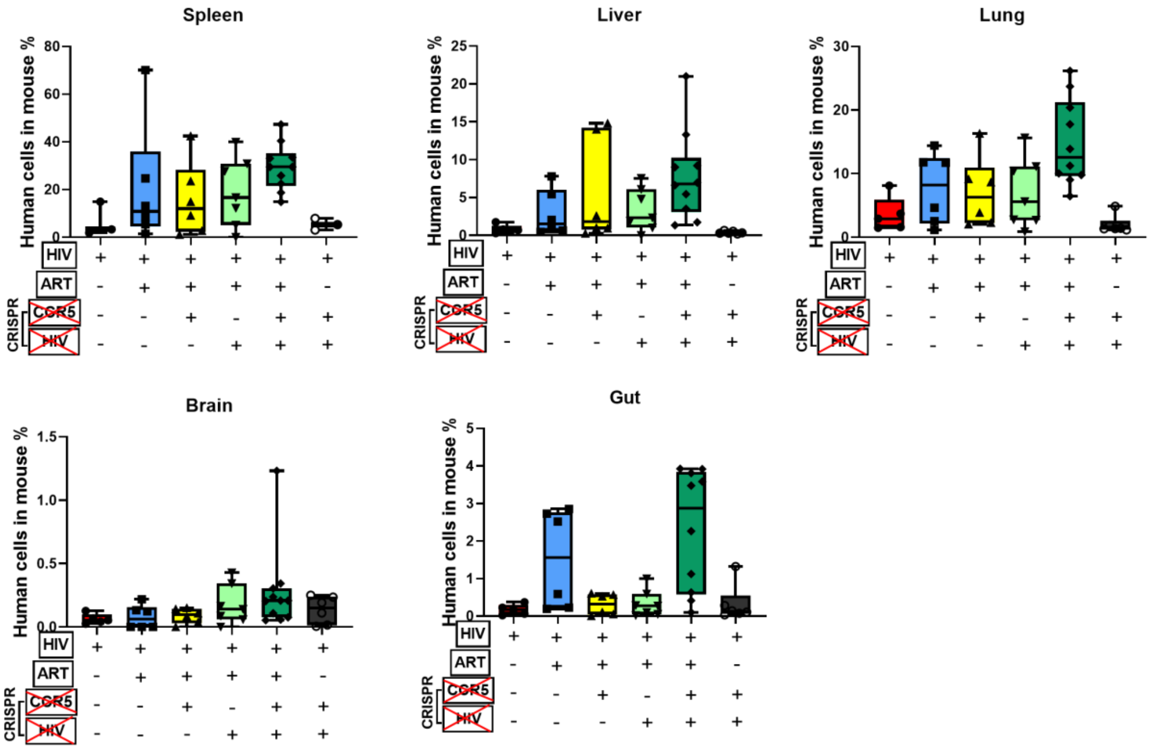

Supporting Data Fig. 19. Bio-distribution of human cells in tissues in CRISPR treated HIV-1 infected humanized mice. Ultrasensitive digital droplet PCR analysis of genomic DNA isolated from the spleen, liver, lung, brain, and gut of humanized mice using primer sets specific to human and mouse TERT. The tissues from LASER ART and dual CRISPR treated group had the highest proportions of human cells compared to single treatments. This indicates the protective/restorative effect of combined treatment on human cells homed in tissues of HIV-1 infected animals, similarly to the one observed for human CD4+ T cells in the blood.

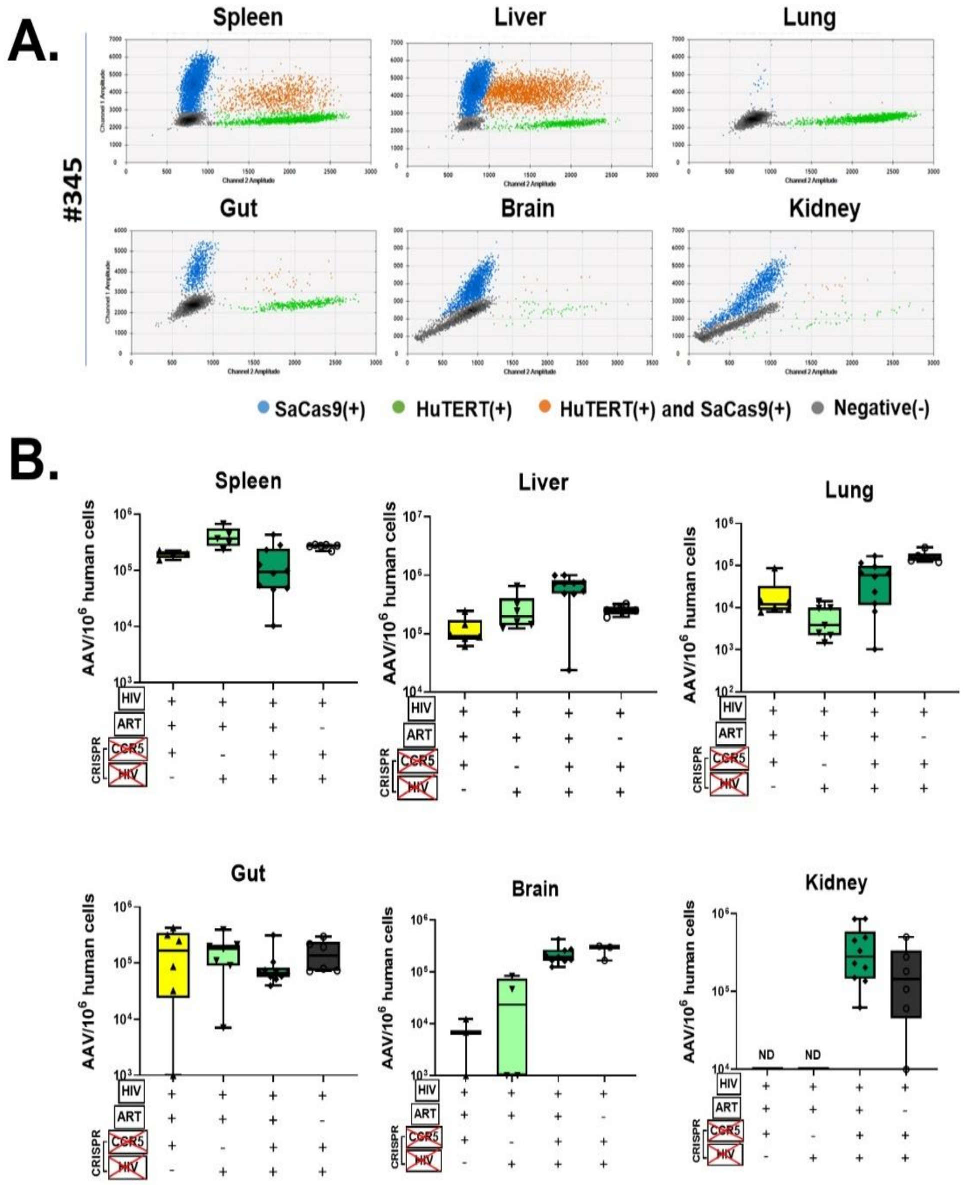

Supporting Data Fig. 20. Bio-distribution of AAV in CRISPR treated HIV-1 infected humanized mouse tissues. Ultrasensitive digital droplet analysis of Cas9 transgene DNA levels in genomic DNA extracted from the spleen, liver, lung, brain, kidney, and gut of AAV-CRISPR treated animals. In **A.** representative images of ddPCR results for each of the tested tissues are shown. **B.** The numbers of double positive AAV-vector (FAM) + human Tert (HEX) droplets for each tissue and experimental group were recalculated to AAV copies per 10^6 human cells.
